# Metabolomics reveals the therapeutic efficacy of liposomal resvida in endometrial cancer through regulating autophagy-related gene expression

**DOI:** 10.1186/s12935-025-04014-3

**Published:** 2025-11-14

**Authors:** Mai O. Kadry, Ahmed Serag, Naglaa M. Ammar

**Affiliations:** 1 Therapeutic Chemistry Department, National Research Centre, Al Bohouth St., Dokki, Egypt; 2 Pharmaceutical Analytical Chemistry Department, Faculty of Pharmacy, Al-Azhar University, Nasr City, Cairo, 11751 Egypt

**Keywords:** Endometrial cancer, Autophagy markers, Metabolomics, mTOR, ERDj-4

## Abstract

Endometrial cancer (EC) is the fourth most abundant gynecological cancer. There is an increase in the incidence of mortality from uterine cancers in the past few decades. A comprehensive systematic study to provide an overview on the relationship between autophagy, metabolomics and the risk of oestradiol valerate (OV) induced endometrial cancer was conducted correlated with the use of liposomal loaded-resvida as an innovate drug delivery system. This article explores how metabolomic technology can offer valuable insights on autophagy molecular aspects in EC by identifying new possible metabolite biomarkers that has the potential to improve the accuracy of diagnosis, prognosis and disease monitoring. Metabolomics approach, included orthogonal partial least squares discriminant analysis (OPLS-DA), thus revolutionizes the management of endometrial cancer. Autophagy described in endometrial cancer, includes the role of HSP-70/C-fos/PTEN/mTOR/ERDj-4/p53 signaling pathways that trigger/inhibit the process and consequently represent a potential molecular targets in therapeutic approaches. Endometrial cancer exhibits a molecular complexity and heterogeneity coherent with histopathologic and metabolomic variability. Multivariate statistical analyses pointed out a noteworthy deviation in serum chemical profiles among control, oestradiol valerate, and Resvida and liposomal-Resvida treated groups. Loading plot guided the selection of differential metabolites, elucidating significant variation in metabolite concentration. Improved characterization of molecular alterations of each histological type provides relevant information about the prognosis and potential response to new liposomal therapies. CA125 as EC biomarker was ameliorated post Resvida (108.7 IU/mL) and liposomal Resvida (82.2 IU/Ml) treatment at P ≤ 0.05 in addition to up regulating autophagy biomarkers including mTOR/Cfos/ERDj-4/ PTEN by 20, 25, 14, and 17 fold change respectively and down regulating p53 protein expression by 0.4 ng/ml at P ≤ 0.05 post OV intoxication with liposomal regimen reflecting the most significant impact in modulating these altered genes. The current metabolomics study is the integration of histopathologic and autophagy molecular factors to improve the diagnosis, prognosis, and treatment of endometrial cancer in coherent with liposomal drug delivery system as a targeted therapy.

## Introduction

Wide evidence, suggested that endometrial cancer originates in the uterine epithelium and is the fourth most common neoplasia for women globally. It can be divided into several histological subtypes, including: (i) endometrioid endometrial cancer (EEC, or Type I); (ii) serious endometrial cancer (SEC, or Type II); (iii) clear cell endometrial cancer (CCEC, or Type II); and (iv) Mixed endometrial cancer and uterine carcinoma (USC). About 70% of diagnosed cases are type I tumors, which are low grade and linked to estrogen stimulation. In contrast, type II tumors are less common, typically high grade, estrogen-independent, clinically aggressive, metastatic, and have a higher chance of relapsing following chemotherapy [58]. Endometrioid adenocarcinoma, the most prevalent histological form of endometrial cancer, is linked to a number of epidemiological risk factors, such as unopposed estrogen use, obesity, diabetes, cigarette smoking, null parity, early menarche, and late menopause. Tumor suppressor PTEN mutations are linked to a lower extent of Cowden syndrome and an increased risk of developing endometrial cancer [5]. During the cycle, endometrial proliferation is a common occurrence. Endometrial cancer develops from normal endometrial proliferation when the endometrium is continuously exposed to estrogen. By raising the level of reactive oxygen species and boosting cellular proliferation, oestradiol valerate (OV) may raise oestrogen levels and cause oxidative stress injury [41].

In the rat model, OV may be associated with an increase in oestrogen levels, which may ultimately result in cellular multiplication and EC [45]. Chemotherapy's clinical efficacy varies, suggesting that new molecular treatments targeting particular cellular mechanisms linked to cell survival and treatment resistance—like autophagy—are needed to increase the success rates of treating endometrial cancer. Through the lysosomal breakdown of cytoplasmic macromolecules and organelles, macroautophagy—a specialized mechanism that preserves cell homeostasis—is triggered in response to cellular stressors such as starvation of amino acids, hypoxia, nutrient deprivation, and metabolic stress in order to extend cell survival. However, because autophagy can be both apoptotic and self-protective it can plays a contentious role in human cancer cells. Treatment resistance may result from the activation of autophagy as a pro-survival tumor response by conventional antitumor therapies such as hormones, chemotherapy, and ionizing radiation. Interestingly, autophagy can cause tumor cells to undergo apoptosis if it persists beyond the point at which cell viability can be reversed [20]. Here, we have examined the mechanisms of autophagy that have been reported in endometrial cancers, including the function of the p53 and mTOR/ERDj-4/PTEN signaling pathways, which either initiate or inhibit the process and are therefore viable molecular targets for therapeutic strategies. Furthermore, recent metabolomic data suggest that autophagy can be regulated by repurposing medications, which could expedite testing and validation and facilitate patient access to the drugs.

In summary, despite the fact that autophagy research in endometrial cancer is uncommon but still ongoing, the results show that novel liposomal loaded formulations show promise for guiding targeted therapies [58]. The primary front-line chemotherapy for endometrial cancer is combination therapy using paclitaxel and carboplatin [4] by their ability to damage DNA and prevent actin microtubules in the cytoskeleton from properly polymerizing. Its clinical efficacy varies, though, suggesting that new molecular treatments targeting particular cellular mechanisms linked to cell survival and resistance to treatment, like autophagy, are needed to increase the success rates of endometrial cancer treatments [22]. Resvida (RSV) is the most appropriate phytoconstituent molecule for cancer since it has a substantially lower negative impact than the others. By altering several pathways, including the phosphoinositol 3 kinase (PI3K)/protein kinase B (AKT)/mammalian target of rapamycin (mTOR) pathway, RSV prevents the start and advancement of cell proliferation. RSV caused EC cells to undergo apoptosis, or programmed cell death, by degrading cell cycle-regulated proteins such as cyclin E, cyclin D1, P53 and proliferating cell nuclear antigen (PCNA) and causing cytochrome c to be released from the mitochondria. A huge advantage has drawbacks, so because RSV is heavily processed by the liver and intestine, it has poor water solubility and low bioavailability. Remarkably, RSV metabolism is also induced by RSV metabolites. Researchers decided to create nano-carriers for improved delivery because of issues with retention time in the body, low aqueous solubility, and poor bioavailability. By using nano-formulations, topical penetration was enhanced by up to 21%, nano-encapsulation was improved, and as a result, bioavailability and permeability were multiplied [36]. In order to improve antiuterine cancer activity, the current work outlines the full profiling of RSV and its nano-formulations. It also discusses metabolomics and macroautophagy techniques, such as the HSP-70/C-fos/PTEN/mTOR/ERDj-3 signaling pathways in OV induced endometrium cancer in rat model.

## Materials and methods

### Chemicals and reagents

Liposomal-resvida was supplied from Avanti Company (USA), whereas OV, resveratrol, Methanol, Acetonitrile, methoxyamine hydrochloride and pyridine were bought from Sigma-Aldrich Co (St. Louis, MO, USA). A stock of OV (30 mg OV in 10 ml of 5% DMSO corn oil) was prepared [45]. mTOR, Cfos and ERDj3 RT-PCR kits product of (Qiagen, USA). CA125, PTEN and P53 ELISA kits were from (Vitrogen Systems, USA). All of the chemicals used have a high analytical rank.

### Animals and treatments

Female Albino rats (No.40: Female) weighing 100–150 g were housed in normal conditions and allowed unrestricted access to food and water for two weeks to acclimate. The experiment was carried out in compliance with the National Institutes of Health's (NIH publication 85–23, 1985) guidelines for experimental animal care and use. The animal facility was of SPF grade.

National Research Center's animal house was utilized herein. The animals were raised under controlled circumstances (20 °C, 55% humidity, and a 12 h light/dark cycle). Animal Care and Use Committee of the National Research Center ethics approval number (04420225) and the US National Institute of Health.

### Experimental design

Proceeding acclimatization, animals were randomely divided into five cages (8 rats).

**G1:** Control group administered corn oil.

**G 2:** Administered OV (IP, 3 mg/kg) for three months (endometrium cancer model) [45].

**G 3:** Administered resvida (10 mg/kg BW) IP post EC induction for 1 month [26].

**G 4:** Administered Liposomal resvida (3 mg/kg BW) IP post EC induction for 1 month [26].

**G 5:** Administered the standard drug for endometrium cancer treatment Keytruda (10 mg/kg BW) IP for 1 month (standard uterine cancer drug).
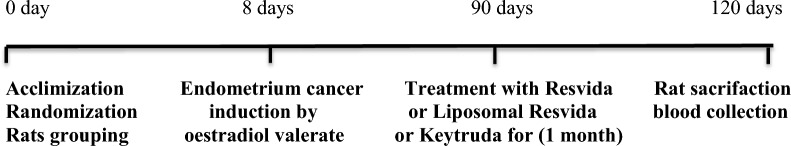


### Blood sampling and tissue preparation

To relieve animal pain, non-invasive procedures were employed, generated a safe and comfortable home for animals, applying 5% CO2 anesthesia during the procedure, and employing analgesic regimens (Isofurane) to dismiss discomfort during the recovery. At the end of experimental period, rats were, slightly anesthetized via carbon dioxide and blood samples were collected from the retro-orbital vein, samples were centrifuged at 5000 rpm for 10 min to obtain serum before being preserved at − 80 °C. Rats were euthanized via cervical dislocation, and uterine tissue was isolated and homogenized in phosphate buffered saline for further biochemical analysis.

### Measured biochemical parameters

**mRNA gene expression of mTOR, C-fos, PTEN, PI3K, AKT and ERDj3:** Initially, total RNA was isolated from uterine tissue using the RNA-easy mini-kit (Qiagen, Germany) and amplified using the RT-PCR kit (Qiagen, USA). The reaction was carried out in a total volume of 20 L of master mix. The thermal profile was as follows: 50^o^ C for 2 min, 95^o^ C for 10 min, and 95^o^ C for 45 to 60 ^o^ C for 30 s, 72^o^ C for 30 s, and 72^o^ C for 10 min (Agilent, MxPro qPCR, Mx3000P). The ∆∆CT fold change method was used to calculate gene analysis. m-TOR, C-fos, PTEN, PI3K, AKT and ERDj3 primers (forward and reverse) illustrated in (Table 1) [29, 33].

**Table 1:** Primers sequence designed for RT-PCR gene expression

Primer Name	Primer sequence
Β- actin	5-CTTTGATGTCACGCACGATTTC-3 5-GGGCCGCTCTAGGCACCAA-3
mTOR	5' AGCATCGGATGCTTAGGAGTGG-3' 5' CAGCCAGTCATCTTTGGAGACC-3'
C-fos	5′-GCC TCG TTC CTC CAG TCC GA-3' 5′-TGC GAT GGA AAG GCC AGC CC-3'
PTEN	5′-GCAGCCATGATGGAAGTT-3′ 3′-ATCGAAATATGCTCAACCTC-5′
ERDj3	5'-AGTAGACAAAGGCATCATTTCCAA-3 5'-CTGTATGCTGATTGGTAGAGTCAA-3'
PI3K	5′-CCA GAC CCT CAC ACT CAG ATCA-3′ 5′-TCC GCT TGG TGG TTT GCT A-3′
AKT	5′-CAT GAA GAG AAG ACA CTG ACC ATG GAAA-3′ 3′-TGG ATA GAG GCT AAG TGT AGA CAC G-5′

### ELISA determination of CA-125, HSP-70 and P53

The activities of CA-125**,** HSP-70 and P53 were measured using ELISA kit (Invitrogen, MN, USA) according to the manufacturer's instructions. The experiment was then evaluated using a quantitative sandwich enzyme immunoassay. Samples were added to the precoated microplate with the selected antibody. The immobilized antibody that bound to CA-125**,** HSP-70 and P53 was detected, following the addition of enzyme-linked secondary antibody specific for CA-125**,** HSP-70 and P53. The color intensity (at 450 nm) was measured (Agient BioTek Microplate reader, Neo2) [28, 32, 50].

### Uterine oxidative stress biomarkers

MDA and BCHE were estimated via kits provided from Randox Company. Further, the absorbance was determined spectrophotometry at 505 nm (V-730 UV–visible spectrophotometer-Jasco Inc.) [23, 30, 33].

### Histopathological examination

Specimen from uterus were collected from all experimental groups, fixed in neutral buffered formalin 10%, washed, dehydrated, cleared and embedded in paraffin. The paraffin embedded blocks were sectioned at 5 micron thickness and stained with Hematoxylin and Eosin for histopathological examination. Histopathological lesion scoring Histopathological alterations of uterus were recorded and scored as, no changes (0), mild (1), moderate (2) and severe (3) changes, the grading was determined by percentage as follows: 50% (severe change) [53]. Lesion score Alteration in uterus were scored according to their severity. Scoring of histopathological alterations in uterus of treated groups Lesions G1:G2: G3: G4: G5—Stratification of endometrial lining epithelium—Cancer and papillary in-folding of endometrial lining epithelium—Cancer of endometrial glands was 0: 3: 0:1: 0 The score system was designed as: score 0 = absence of the lesion in all rats of the group (n = 5), score 1 = (50%) and score 3 = (80%).

### Metabolomics analysis

#### Sample preparation

Serum samples (n = 6) for each group were processed for metabolite analysis according to Aliaa et al. [19]. Briefly, 300 µL of ice-cold extraction solvent (acetonitrile) was added to each serum sample, followed by freezing for 15 min. All samples were subsequently centrifuged at 20,000 g at 4 °C for 15 min. The resulting supernatant was transferred to GC–MS vials and dried under nitrogen gas using a speed vacuum concentrator prior to derivatization.

#### Derivatization steps

Following complete dryness of the extracts under nitrogen, 50 µL of methoxyamine solution (15 mg/mL methoxyamine hydrochloride in pyridine) was added to each dried sample and incubated for 60 min at 70 °C. The derivatization process was then completed by adding 100 µL of MSTFA containing 1% TMC, followed by incubation at room temperature for 60 min.

#### GC–Ms condition

GC–MS system at National Research Center, Cairo, Egypt using thermo mass spectrometer detector (ISQ Single Quadrupole Mass Spectrometer) and HP-5 M column (30 m × 0.25 mm internal diameter and 0.25 mm film thickness). The helium carrier was used at a flow rate of 1.0 ml/min with injection volume 1 micro at a split mode (1:10) and temperature program 80 ^ο^C for 2 min; rising at 5 ^ο^C /min to 300 ^ο^C and held for 5 min. The electron ionization (EI) of Mass spectra was optimized at 70 eV with a spectral range 25–550. The solvent delay time started from 3.7.

#### Transmission electron microscopy (TEM)

Transmission electron microscopy, or TEM, is a common method for examining the size and shape of liposomes in order to characterize liposomal Resvida. A sample is prepared by putting liposomal resveratrol onto a copper grid, frequently staining it with a negative stain such as phosphotungstic acid, drying it, and then looking at it under a TEM to see the liposomal structures.

### Statistical analysis

#### Multivariate metabolic analysis

The netcdf files generated from GCMS analysis of serum metabolites were analyzed using MS-Dial software v4.90. First the files were converted to abf format compatible with MS-Dial. Then, the peaks were aligned, deconvoluted, and annotated using the NIST 17 library. Peaks were filtered based on quality control criteria, and the final data matrix was exported to the SIMCA software for multivariate statistical analysis. Principal Component Analysis (PCA) and Partial Least Squares-Discriminant Analysis (PLS-DA) were performed to study the metabolic changes between the groups (n = 6). Orthogonal Partial Least Squares-Discriminant Analysis (OPLS-DA) was applied to identify the important metabolites that discriminate between the groups. ROC curves were plotted to assess the diagnostic potential of the developed models. For the statistical analysis, GraphPad Instat 3 (GraphPad Software Inc., San Diego, CA, USA) was utilized. SPSS 16 was used to analyze the data using one-way ANOVA, followed by a post-hoc Tukey's test. P-values of less than 0.05 were considered statistically significant.

## Results

### Modulating protein expression of CA-125

**Fig. 1:**
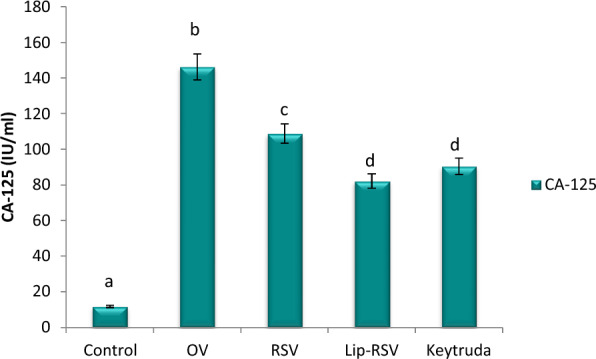
Impact of RSV and Lip-RSV on CA-125 protein expression post OV induced endometrium cancer. Data are expressed as means ± SEM (n = 8), P < 0.05. Groups having different letters are considered significantly different, while, groups having similar letters are not significantly different from each other

The protein expression of uterine cancer biomarker CA-125 was dramatically increased by mean value of 146.23 IU/mL post OV adminstration. Meanwhile, resveratrol, Liposomal-resveratrol, and Keytruda considerbly improved CA-125 protein expression when compared to the control value, with Liposomal-resvida outperforming the others (Fig. 1).

### Modulating autophagy biomarkers; mTOR, ERDj3 and C-Fos gene expression

**Fig. 2:**
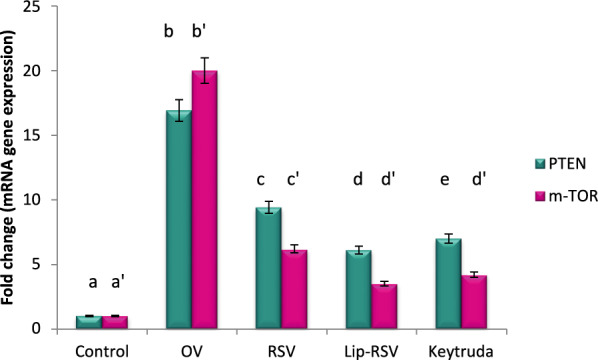
Impact of RSV and Lip-RSV on PTEN and mTOR gene expression post OV induced endometrium cancer. Data are expressed as means ± SEM (n = 8), P < 0.05. Groups having different letters are considered significantly different, while, groups having similar letters are not significantly different from each other. Β-actin was used as reference gene

**Fig. 3:**
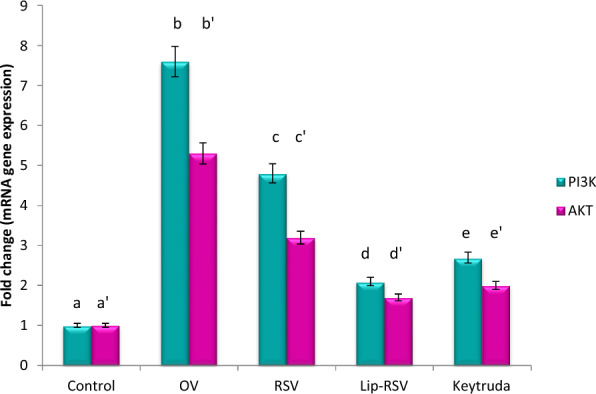
Impact of RSV and Lip-RSV on PI3K and AKT gene expression post OV induced endometrium cancer. Data are expressed as means ± SEM (n = 8), P < 0.05. Groups having different letters are considered significantly different, while, groups having similar letters are not significantly different from each other. Β-actin was used as reference gene

**Fig. 4:**
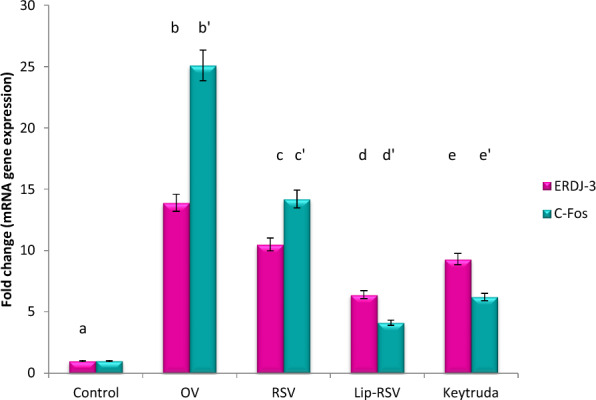
Impact of RSV and Lip-RSV on ERDj3 and C-fos gene expression post OV induced endometrium cancer. Data are expressed as means ± SEM (n = 8), P < 0.05. Groups having different letters are considered significantly different, while, groups having similar letters are not significantly different from each other. Β-actin was used as reference gene

The autophagy biomarkers mTOR and ERDj3 and apoptotic and cell survival biomarkers C-Fos, were dramatically upregulated by about 20,13.9 and 25 fold change respectively post OV administration, indicating endoplasmic reticulum stress and apoptotic stimulation. Meanwhile, resveratrol, Liposomal-resveratrol, and Keytruda considerbly modified these altered genes when compared to the control value at P ≤ 0.05, with Liposomal-resvida having the greatest impact (Figs. 2, 3 and 4).

### Modulating cell survival biomarkers; PI3K, AKT and PTEN gene expression

The cell survival biomarkers AKT, PI3K and PTEN were dramatically upregulated by about 5.3, 7.6 and 16.9 fold change post OV administration as compared to the control value, indicating endoplasmic reticulum stress and apoptotic stimulation. Meanwhile, resveratrol, Liposomal-resveratrol, and Keytruda considerably modified these altered genes when compared to the control value, with Liposomal-resvida having the greatest impact (Figs. 2, 3 and 4).

### Modulating protein expression of HSP-70 and P53

**Fig. 5:**
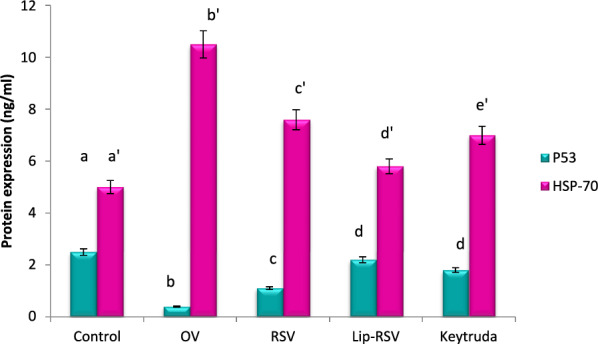
Impact of RSV and Lip-RSV on P53 and HSP-70 protein expression post OV induced endometrium cancer. Data are expressed as means ± SEM (n = 8), P < 0.05. Groups having different letters are considered significantly different, while, groups having similar letters are not significantly different from each other

OV significantly increased HSP-70 by a mean value 10.5 ng/ml and decreased P53 protein expression by mean value 0.4 ng/mL. Meanwhile, resveratrol, Liposomal-resveratrol, and Keytruda greatly improved these changed proteins when compared to the control value, with Liposomal-resvida outperforming the others (Fig. 5).

### Modulating oxidative stress biomarkers

**Fig. 6:**
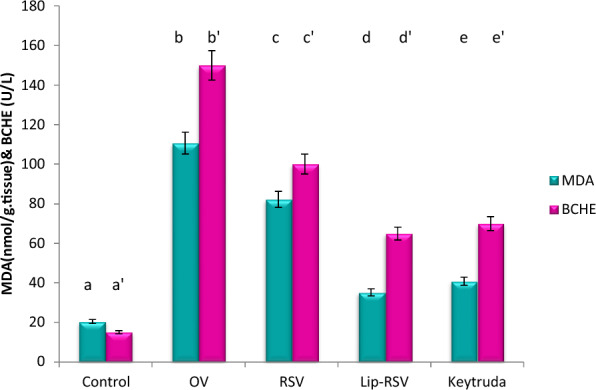
Impact of RSV and Lip-RSV on MDA and BCHE levels post OV induced endometrium cancer. Data are expressed as means ± SEM (n = 8), P < 0.05. Groups having different letters are considered significantly different, while, groups having similar letters are not significantly different from each other

OV increased the oxidative stress biomarker MDA while decreasing the anti-oxidative stress biomarker BCHE, indicating an oxidative stress and redox imbalance. Meanwhile, resveratrol, Liposomal-resveratrol, and Keytruda considerably modified these changed biomarkers as compared to the control, with Liposomal-resvida having the greatest impact (Fig. 6).

### Histopathological findings

**Fig. 7:**
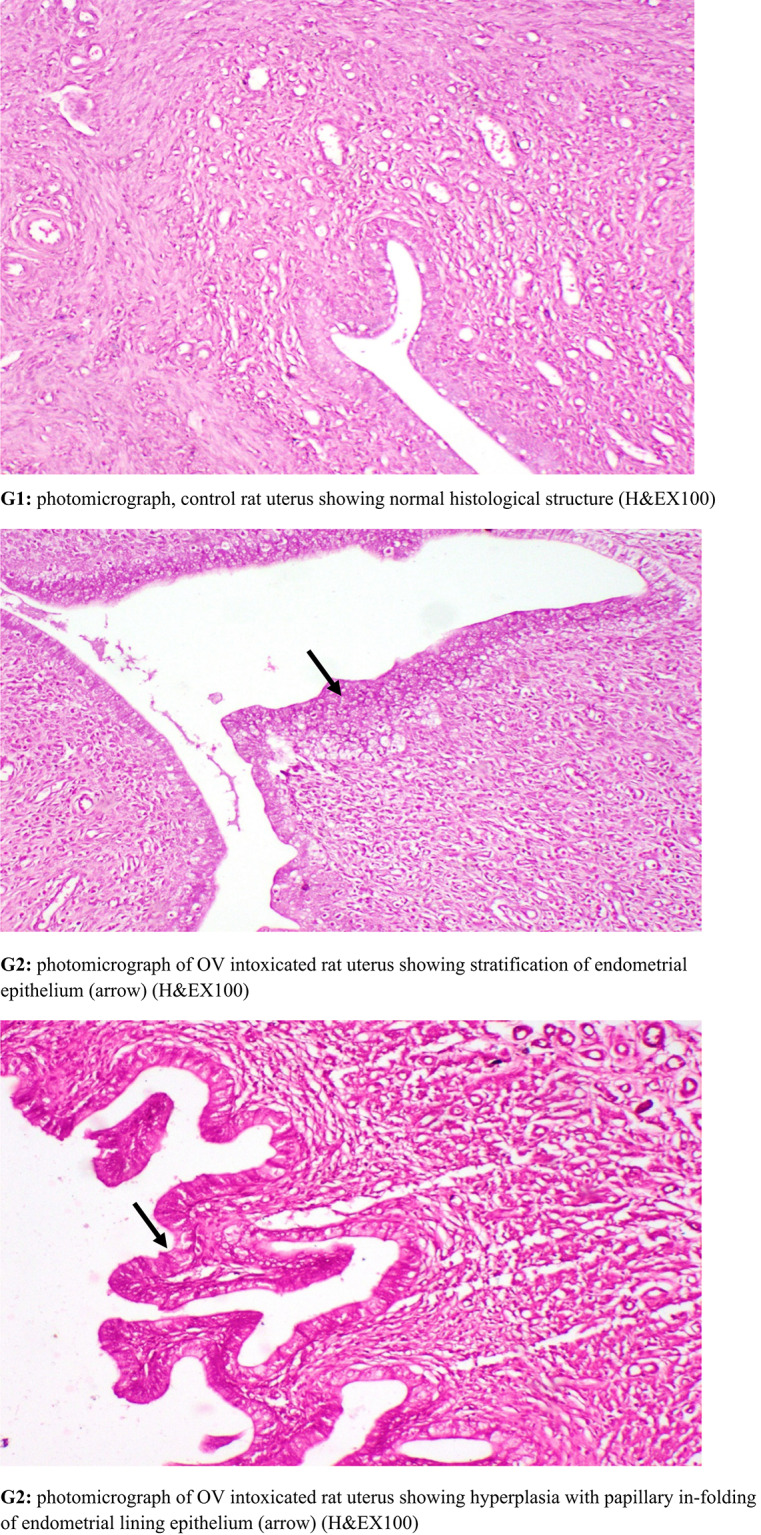

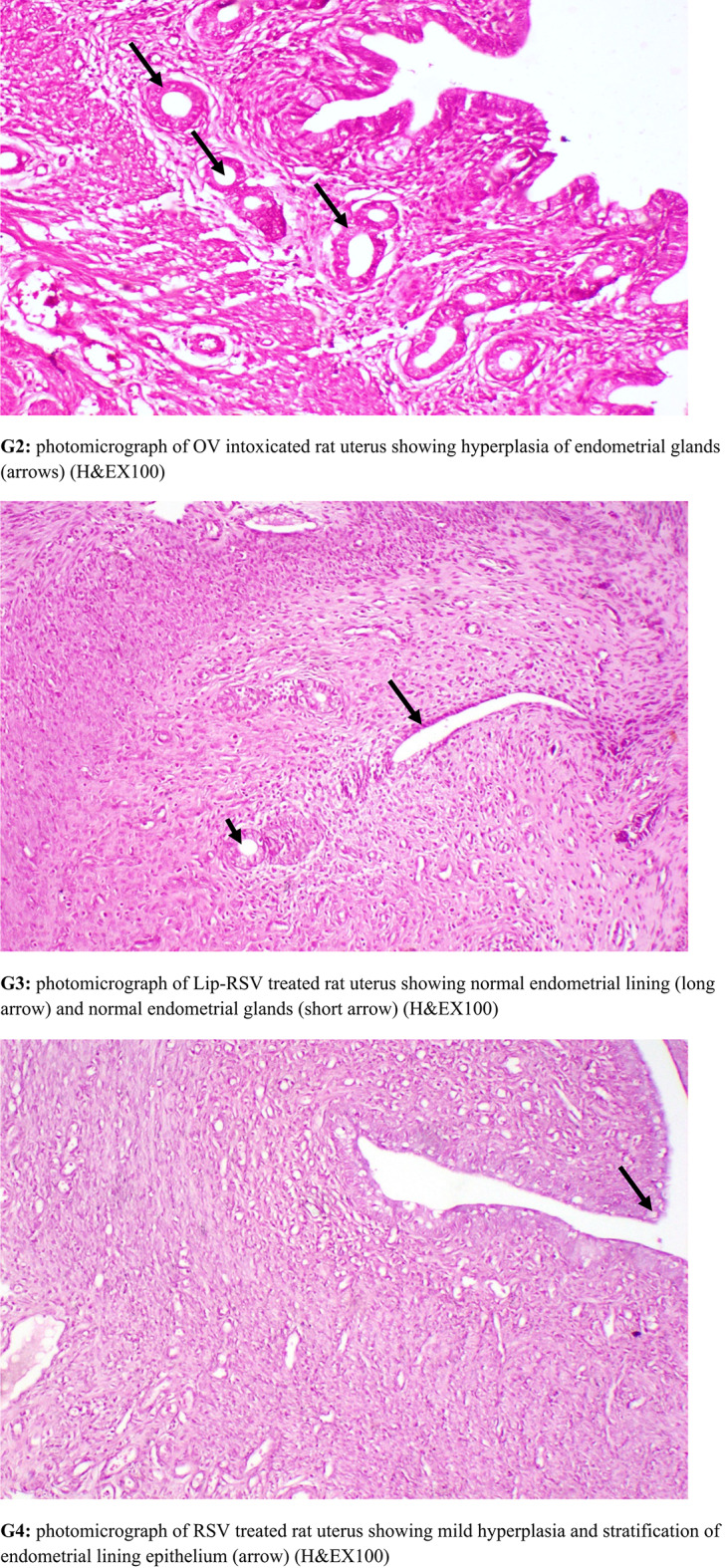

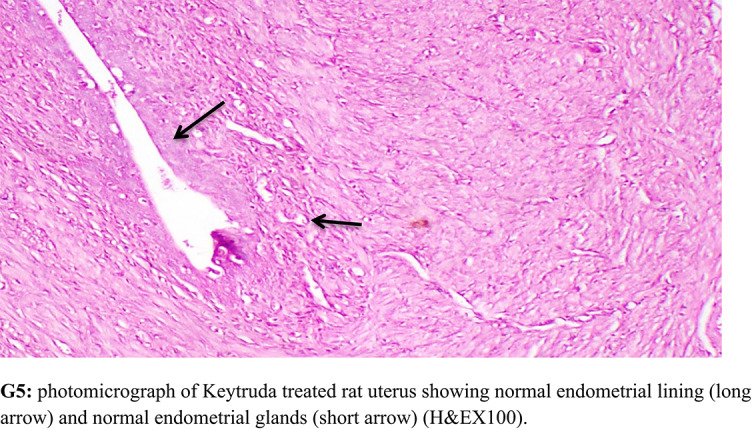
Histopathological investigations for **G1:** control group revealing normal uterine lining epithelium and uterine glands. **G2:** OV group revealed cancer and stratification of endometrial lining epithelium, cancer and papillary in-folding of endometrial epithelium and cancer with increased number of endometrial glands. **G3:** Lip-RSVgroup showed nearly normal structure of uterine lining epithelium and uterine glands. **G4:** RSV group showed mild cancer of endometrial epithelium with normal endometrial glands. **G5:** Keytruda group uterus showing normal and stratification of endometrial lining epithelium

OV group revealed cancer and stratification of endometrial lining epithelium, cancer and papillary in-folding of endometrial epithelium and cancer with increased number of endometrial glands. Meanwhile, Lip-RSVgroup showed nearly normal structure of uterine lining epithelium and uterine glands. RSV group showed mild cancer of endometrial epithelium with normal endometrial glands. Keytruda group revealed normal and stratification of endometrial lining epithelium (Fig. 7).

### Metabolic analysis

#### Serum metabolites profiling in OV, RSV and Lip-RVS via GC–MS and data analysis between groups

##### Multivariate metabolic analysis

**Fig. 8:**
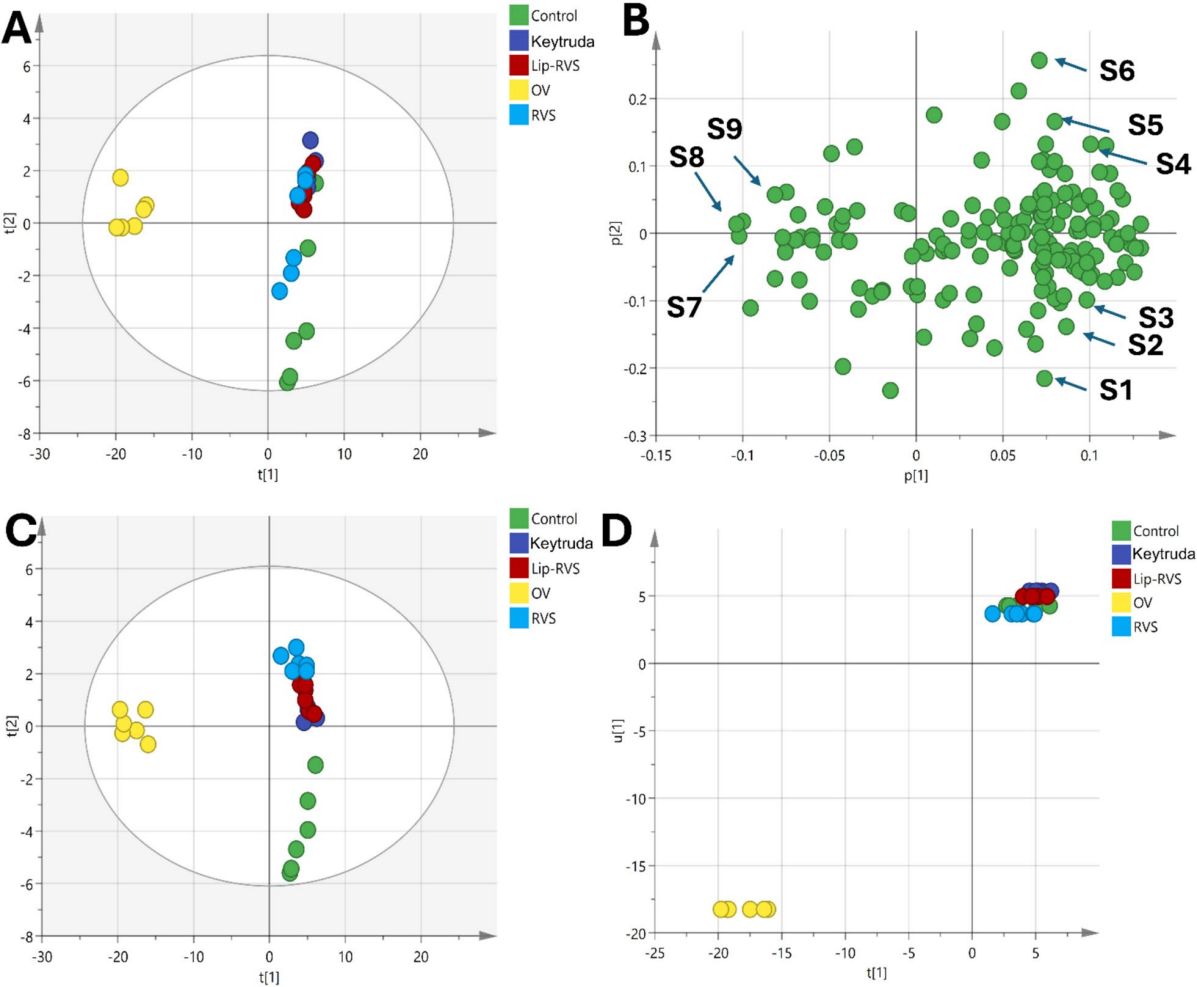
**A** PCA score plot shows distinct separation between the OV group and other groups along the first principal component, accounting for significant metabolic changes. **B** PCA loading plot identifies abundant metabolites in the control and treated groups. **C** PLS-DA score plot validates separation among the groups with satisfactory model performance (R2Y = 0.797, Q2 = 0.635), showing clear clustering. **D** Class inner relations plot highlights the closeness of treatment groups to the control, with the Lip-RVS group being the most similar to the control group. The identified metabolites as follow: proline (S1), serine (S2), indole-3-propanoic acid (S3), butanoic acid (S4), leucine (S5), aspartic acid (S6), γ-palmitolactone (S7), hexadecanoic acid (S8), octadecanoic acid (S9)

Multivariate data analysis revealed specific metabolic signatures associated with OV and administration of resveratrol, Liposomal-resveratrol, and Keytruda. A total of 175 metabolites were detected and survived quality control filtering with RSD < 30%. Unsupervised PCA was performed to visualize the global variation in the data. The PCA scores plot showed a clear separation between the OV and the other groups along the first principal component, indicating significant metabolic perturbations in the OV group relative to the control with the first two components explaining 75.6% of the variation (Fig. 8A). The loading plot identified the metabolites that increased in the OV group such as γ-palmitolactone, oxymetholone and propofol (Fig. 8B). In contrast, several metabolites were abundant in control and treatment groups, such as proline, serine, leucine, phenylalanine, ornithine, glycine, aspartic acid, propanoic acid, phosphoric acid and urea (Fig. 8B). Consequently, supervised PLS-DA model separating the five treatment groups was constructed and validated, with a satisfactory R2Y and Q2 of 0.797 and 0.635 respectively using three latent variables. The score plot showed clear clustering of the groups, corroborating the PCA findings (Fig. 8C). Additionally, the class inner relations plot revealed the closeness between the treatment groups and the control, with the Liposomal-resvida group being the closest to the control (Fig. 8D).

**Fig. 9:**
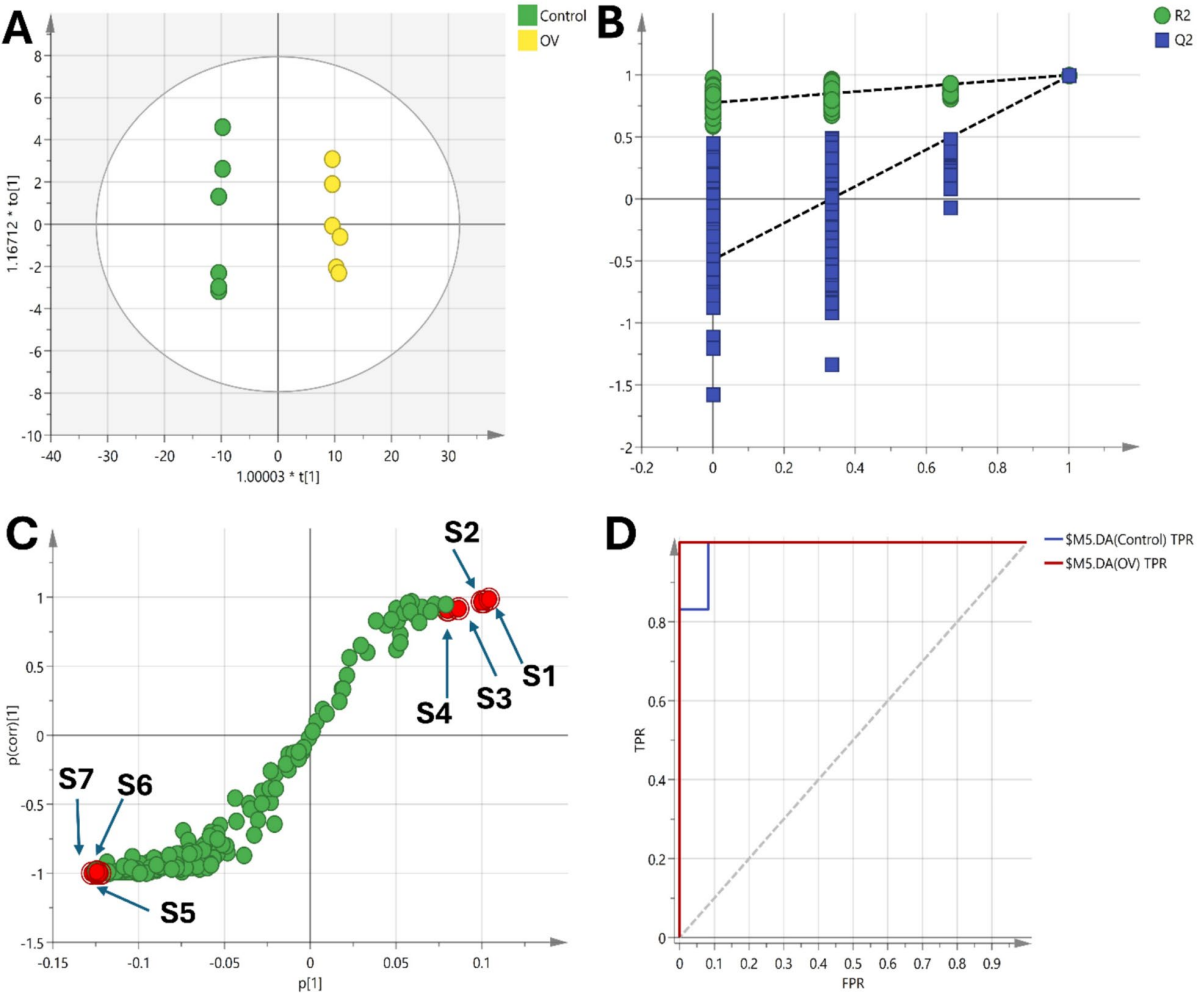
**A** Scores plot of the OPLS-DA model showing clear separation between the OV and control groups along the predictive component, indicating significant metabolic differences. **B** Permutation test validating the model's reliability, with a negative intercept supporting the robustness of the OPLS-DA model. **C** S-plot highlighting the metabolites driving the separation between the groups, including increased levels of γ-palmitolactone (S1), hexadecanoic acid (S2), octadecanoic acid (S3) and propofol (S4) were increase in OV group while proline (S5), serine (S6), and indole-3-propanoic acid (S7) were elevated in the control group. **D** ROC curve of the OPLS-DA model demonstrating excellent diagnostic performance with an AUC of 0.986 for distinguishing OV from control

Pairwise OPLS-DA models were applied to identify the metabolites that were significantly altered in the OV group compared to the other groups. The first OPLS-DA model compared OV vs control had a satisfactory R2Y and Q2 of 0.845 and 0.998 respectively (Fig. 9). A clear separation between the two groups was observed in the scores plot along the predictive component, indicating substantial metabolic differences (Fig. 9A). The permutation test confirmed the validity of the model with negative intercept value (Fig. 9B). The metabolites that contributed most to the separation were identified based on the S plot that modelled the covariance and correlation structures between the X and Y matrices (Fig. 9C). These included increased levels of oxymetholone, γ-palmitolactone, propofol, hexadecanoic acid and octadecanoic acid in the OV group compared to control. On the hand, proline, serine, leucine, phosphoric acid and 1 h indole 3-propanoic acid were increased in the control group. ROC curves were plotted to evaluate the diagnostic potential of the developed OPLS-DA model. The area under the curve for differentiating OV from Control was 0.986, indicating excellent diagnostic accuracy (Fig. 9D).

**Fig. 10:**
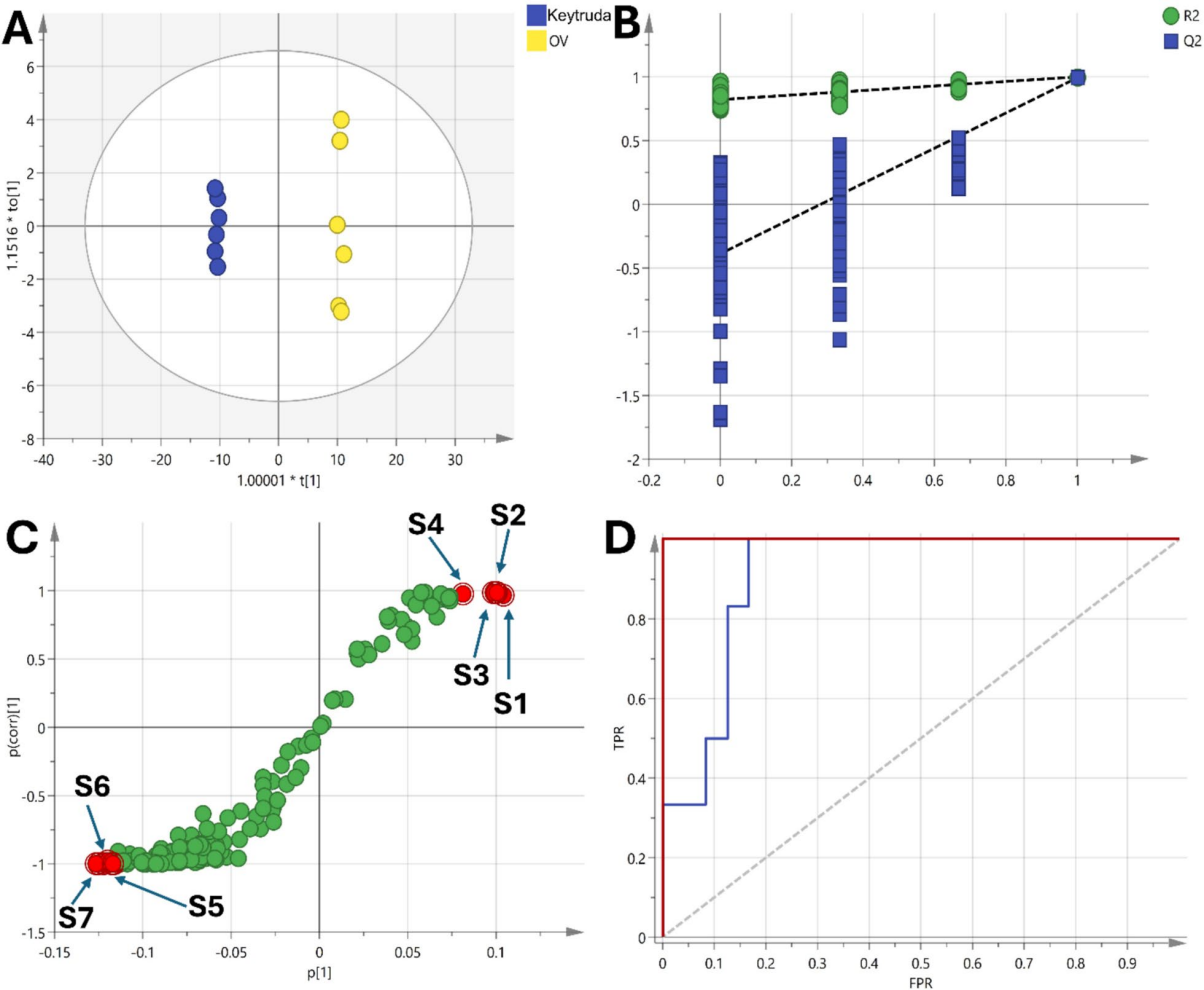
**A** Scores plot of the OPLS-DA model showing clear separation between the OV and the standard drug-treated group along the predictive component, indicating significant metabolic differences. **B** Permutation test validating the model's reliability, with a negative intercept supporting the robustness of the OPLS-DA model. **C** S-plot highlighting the metabolites driving the separation between the groups, including increased levels of γ-palmitolactone (S1), oxymetholone (S2), octadecanoic acid (S3) and propofol (S4) were increase in OV group while phosphoric acid (S5), aspartic acid (S6), and Propanoic acid (S7) were elevated in the standard drug-treated group. **D** ROC curve of the OPLS-DA model demonstrating excellent diagnostic performance with an AUC of 0.917 for distinguishing OV from standard drug-treated group

**Fig. 11:**
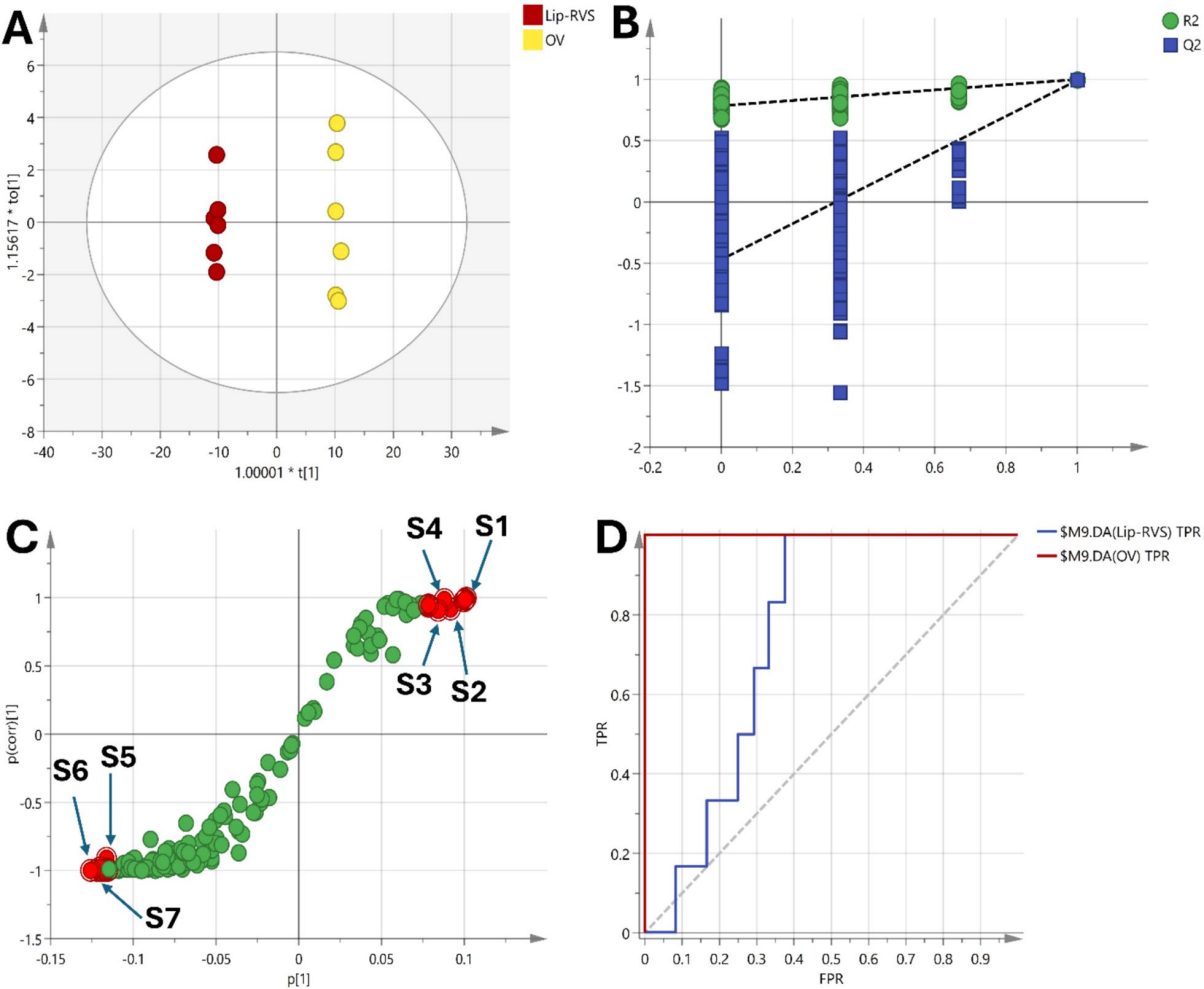
**A** Scores plot of the OPLS-DA model showing clear separation between the OV and the Lip-RSV-treated group along the predictive component, indicating significant metabolic differences. **B** Permutation test validating the model's reliability, with a negative intercept supporting the robustness of the OPLS-DA model. **C** S-plot highlighting the metabolites driving the separation between the groups, including increased levels of γ-palmitolactone (S1), oxymetholone (S2), octadecanoic acid (S3) and propofol (S4) were increase in OV group while phosphoric acid (S5), aspartic acid (S6), and glycine (S7) were elevated in the Lip-RSV-treated group. **D** ROC curve of the OPLS-DA model demonstrating excellent diagnostic performance with an AUC of 0.750 for distinguishing OV from Lip-RSV-treated group

**Fig. 12:**
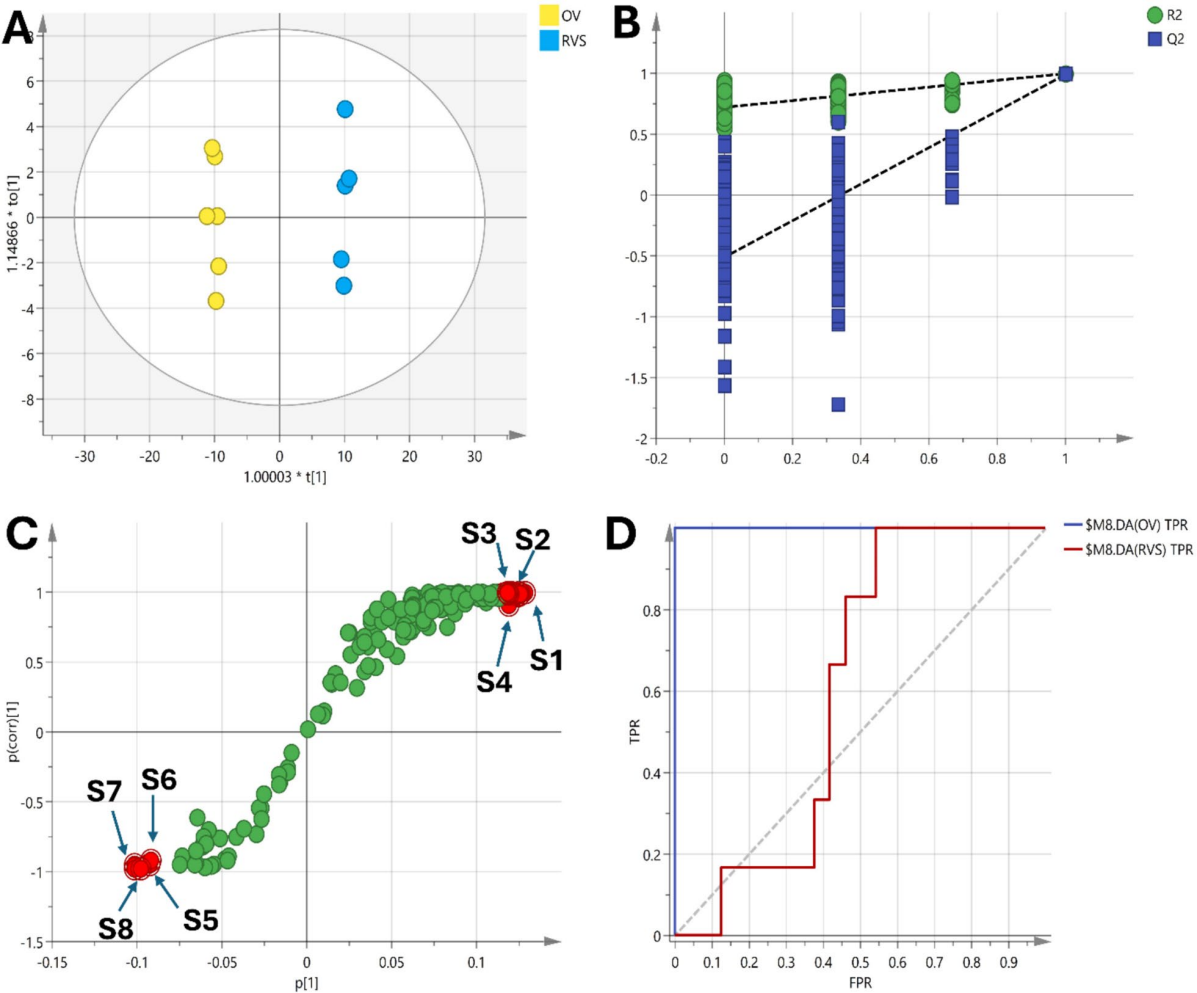
**A** Scores plot of the OPLS-DA model showing clear separation between the OV and the RSV-treated group along the predictive component, indicating significant metabolic differences. **B** Permutation test validating the model's reliability, with a negative intercept supporting the robustness of the OPLS-DA model. **C** S-plot highlighting the metabolites driving the separation between the groups, including increased levels of glycine (S1), phenylalanine (S2), urea (S3) and serine (S4) were elevated in the RSV-treated group while γ-palmitolactone (S5), oxymetholone (S6), octadecanoic acid (S7) and propofol (S8) were increase in OV group. **D** ROC curve of the OPLS-DA model demonstrating lower diagnostic performance with an AUC of 0.611 for distinguishing OV from RSV-treated group

Similarly, OPLS-DA models comparing the OV group with the treatment groups were constructed (Figs. 10, 11, 12). The OV vs RSV model had an R2Y and Q2 of 0.857 and 0.997 respectively, while the OV vs Lip-RSV and the OV vs Keytruda models had R2Y and Q2 values of 0.852, 0.999 and 0.863, 0.999 respectively, all indicating robust model performance. The scores plots showed clear separation of the groups along the predictive component, corroborating the metabolic differences (Figs. 10A, 11A & 12A). Validation through permutation testing confirmed the validity of the models (Figs. 10B, 11B & 12B). The metabolites that contributed most to the class separations were identified from the corresponding S-plots (Figs. 10C, 11C & 12C). Interestingly, the ROC curves for differentiating OV from the treatment groups showed different degrees of diagnostic accuracy, with the OV vs Keytruda model achieving the highest AUC of 0.917 (Fig. 10D), followed by OV vs Lip-RSV (0.750) (Fig. 11D) and OV vs RSV (0.611) (Fig. 12D), underscoring the superior metabolic modulation by liposomal-resvida compared to the non-formulated counterpart.

**Figure:**
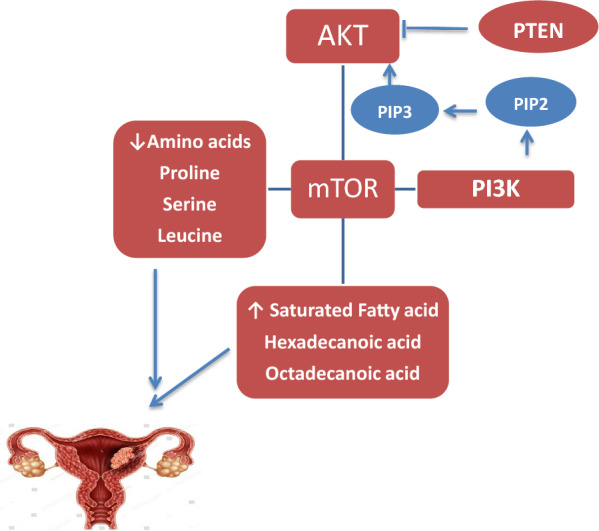
**Schematic diagram:** representing the correlation between PI3K and stimulating the phosphorylation of PIP2 to PIP3 that further activate AKT signaling promoting autophagy biomarker mTOR signaling that affect lipid metabolism and stimulate saturated fatty acid (hexagecanoic acid and Octadecanoic acid) accumulation on the other hand reduce amino acids (Proline, seine and leucine) formation, combining together these factors endometrium cancer was triggered

#### Transmission electron microscopy

**Fig. 13:**
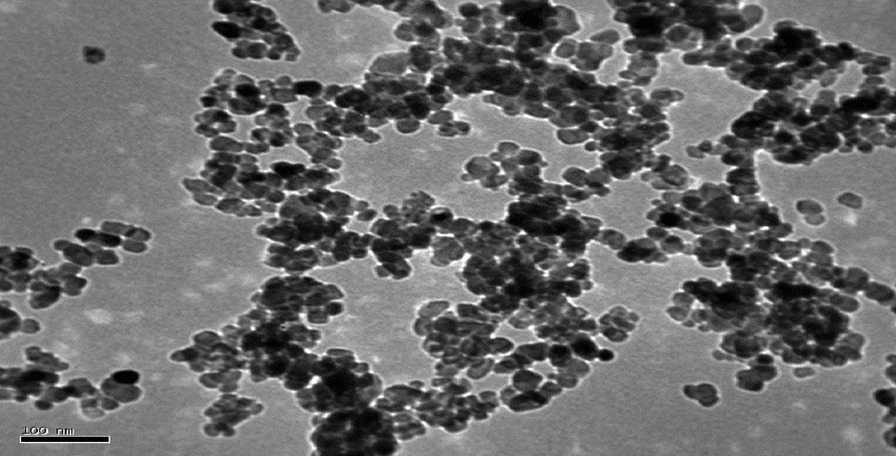
Transmission electron microscopy (TEM) image for liposomal Resvida (Scale bar 100 nm)

TEM revealed spherical structure of Lip-RVS and diameter < 80 nm, the observed zeta potential was—70 mV and the encapsulation efficiency was 81.9% (Fig. 13).

## Discussion

Macroautophagy is an essential component of cellular functions where cytoplasmic components are removed by the lysosome. Autophagy helps maintain cellular homeostasis by lowering the quantity of damaged proteins and organelles in cells. Furthermore, numerous diseases, including cancer, metabolic syndrome, and neurodegenerative diseases, can result in autophagy dysfunctions. Autophagy can ensure cell survival, but under some circumstances, it can also cause cell death [7].

Post OV intoxication, the current investigation elucidated a considerable up regulation in mTOR and PTEN gene expression and a significant down regulation in P53 protein expression. In contrast, therapy with RVS and Lip-RVS significantly controlled these aberrant genes, with liposomal regimen being superior. According to Zhu et al. [64], OV triggered the production of reactive oxygen species (ROS), peroxide lipids, inhibit p53 signaling, stop the cell cycle, and activate both intrinsic and extrinsic apoptotic pathways in EC. According to Lin et al. [38], OV triggered the PI3K/AKT/mTOR pathway, which in turn altered the autophagy flux in the endometrial cancer cell line Ishikawa. Furthermore, the level of autophagosomes increased following OV intervention. mTOR and AKT kinases control this fine tuning of autophagy gene expression. Furthermore, the expression of important genes for autophagy activation (LKB1, ULK1/2) and autophagosome maturation (ATG4, ATG7, and ATG10) were regulated via the p53 transcription factor [16]. P53 played a crucial role in converting MSH2 signals into the autophagic pathway. P53 has an unexpected role in regulating autophagy through the induction of apoptosis [42]. MHY2256, an inhibitor of SIRT protein, has been demonstrated to have anticancer effects by causing p53 protein to be acetylated in estrogen-positive EC cells and increased late apoptosis and markedly decreased tumor development. The cell cycle regulators p21 and acetylated P53 may have a role in the mechanism of autophagy activation, by increasing AMPK and tuberous sclerosis complex 2, P53 facilitated autophagy-regulation, and suppress mTOR [15].

According to Kadry and Abdel-Megeed [27], PI3K/AKT/PTEN/mTOR signaling plays a crucial role in controlling gene transcription, invasion, proliferation, and cell survival. It also plays a key role in metabolism by controlling enzymes such as Glyoxalase 2 and Glyoxalase 1, which accelerate the development of cancer. The PI3K/AKT pathway is downstream of the master regulator mTOR, a serine/threonine kinase with essential functions in autophagy. In healthy cells, mTOR inhibits autophagy through phosphorylating mAtg13 and ULK1; but, upon stress stimulation, mTOR kinase is inhibited [38]. mTOR pathway activation reversed the effects of autophagy and cancer hallmarks in endometrial cancer [6]. Progestin-resistant cells developed in vitro demonstrated enhanced cell proliferation and resistance to autophagy as demonstrated by activation in the PI3K/AKT/mTOR signaling pathway. The molecular characteristics of endometrial carcinomas revealed distinctive PTEN, PIK3CA, PIK3R1 and K-RAS mutation. 11% of type I carcinomas and over 90% of type II carcinomas possess TP53 mutations. In endometrial cancer, somatic mutations were found in several autophagy genes, including mTOR/ERDj and ATG7. Common mTOR mutation, such as hotspot mutations in S2215Y and C1483F, were observed [18].

Post OV intoxication, the current study elucidated that C-Fos gene expression was dramatically up regulated. In contrast, RVS and Lip-RVS therapy significantly down regulated this deviated gene, demonstrating the advantages of the liposomal regimen. c-FOS, a transcription factor belonging to the AP-1 family, controls genes involved in angiogenesis, invasion, motility, proliferation, differentiation, and apoptosis, among other aspects of malignant development [39]. Furthermore, in mouse mammary tumor cells, c-FOS has been shown to affect cell–cell and cell–matrix adhesion. Although C-Fos was first identified as an oncogene, it was linked to a positive prognosis for patients with endometrial cancer. Proliferation, migration, and apoptotic potential of c-FOS-overexpressing clones were observed using stable transfectants of SKOV3 and OVCAR8 cells [46]. c-FOS overexpression significantly accelerated the formation of tumors after injection into SCID mice, resulting in more circulating tumor cells and lung metastases. In c-FOS-overexpressing OvCa cells, adhesion to extracellular matrix components (collagen I, IV), E-selectin, endothelial, and mesothelial cells was decreased. This was consistent with the deregulation of adhesion proteins and glycosylation enzymes. c-FOS may affect OvCa progress by altering OvCa cells' adherence to peritoneal surfaces in addition to its pro-apoptotic action and impact on programmed cell death. A well-differentiated phenotype, more metastases than primary tumors, and an independent positive prognostic sign are all associated with high c-FOS expression in ovarian cancer (OvCa) [44].

There was also a notable increase in HSP-70 protein expression following OV intoxication. Treatment with RVS and Lip-RVS dramatically decreased HSP-70 protein expression with the superiority of liposomal regimen, Because HSP70s are widely distributed in cancer, they give malignant cells a selective edge by inhibiting several apoptotic pathways, controlling necrosis, evading the cellular senescence program, disrupting tumor immunity, encouraging angiogenesis, and aiding metastasis. The concept of cancer "addiction" to HSP70 is explained by the direct involvement of HSP-70 in the majority of cancer hallmarks, which closely correlates HSP70 expression with tumor development and survival. Through its catalytic cycle, HSP70 functions in a variety of states, indicating that it possess multi-function in malignant cells. In the clinical setting, tumor cells release HSP-70 in large quantities into the extracellular milieu, which possess various impacts on patient survival. Clinical trials evaluated a number of HSP70-based immunotherapy strategies [61]. Both innate and adaptive immunological responses can be triggered by HSP70 [62]. In this sense, extracellular HSP70 (eHSP70) activated T regulatory cells, which led to the upregulation of transforming growth factor-β (TGF-β) and IL-10 cytokines and the downregulation of interferon-γ (IFN-γ) and TNF-α. By activating nuclear factor kappa B (NF-kB), the interaction of eHSP70 with antigen-presenting cells resulted in the production of inflammatory cytokines such TNF-α, IL-1β, IL-6, and IL-12. Through its interaction with Tumor necrosis factor receptor-1 (TNFR1), the Tag7-HSP70 complex causes autophagy and lysosomal membrane permeabilization. Both intrinsic and extrinsic apoptotic pathways were clearly impacted by HSP70. According to reports, the HSP70-CHIP complex facilitates the proteasomal degradation of apoptosis signal-regulating kinase 1 (ASK1) in the model of TNF-α-induced apoptosis, which inhibits P53 and c-Jun N-terminal kinase (JNK) [21]. Apoptosis-inducing factor (AIF), which is necessary for DNA fragmentation, is inhibited by HSP70. In addition to apoptosis, HSP70 is crucial for autophagy and necrosis. HSP70 was discovered to be present in the lysosomes of cancer cells, in contrast to normal cells, HSP70 can stabilize lysosomal membranes, which enables cancer cells to evade cell death [12].

According to the current study, ERdj3 was highly up regulated post OV intoxication, while this gene was dramatically downregulated after receiving RVS and Lip-RVS, with liposomal regimen being preferable. ERdj3 a component of unassembled Ig heavy chain BiP complex, is a soluble ER luminal glycoprotein. ER stress transcriptionally up-regulated ERdj3, which was widely expressed and most abundant in secretory tissues. ERdj3 is a cofactor for BiP's roles in protein folding and assembly, as evidenced by its direct binding to several unfolded and mutant secretory pathway protein substrates in addition to its connection with Ig heavy chains. Through a highly conserved mechanism involving ATP-dependent regulation of HSP70 binding protein substrates, the HSP70 chaperoning pathway controls proteostasis [52]. Numerous proteostasis processes, such as protein folding, breakdown, and disaggregation, polypeptide translocation across membranes, and the control of stress-responsive signaling cascades, are mediated by these HSP70 chaperones. Among ER HSP40s, the soluble Type II HSP40 co-chaperone ERdj3/DNAJB11 is one of the most prevalent. ERdj3 play various roles in regulating extracellular proteostasis and the ER. As a conventional HSP40 co-chaperone in the ER, ERdj3 binds to misfolded proteins and transports them to the ER HSP70 BiP for ATP-dependent chaperoning. By this method, ERdj3 controls the folding or breakdown of secretory proteins, such as glucocerebrosidase, epithelial sodium channel, and immunoglobulin light and heavy chains [14]. Furthermore, by interacting with the Sec61 translocon, ERdj3 controls ER permeability and protein translocation into the ER. ERdj3, on the other hand, is released into the extracellular environment in response to ER stress and acts as an ATP-independent extracellular chaperone to stop secreted proteins from misfolding and/or aggregating thus controlling autophagy [34].

The metabolomics analysis performed via gas chromatography—mass spectrometry (GC–MS) confirmed the formely mentioned genomic analysis and revealed a noteworthy elevation in L-aspartic acid, proline, 5-Oxoproline, propionic acid, heptanedioic acid, phenyl alanine, isoleucine, leucine, serine and glycine in rats treated with RVS and Lip-RVS versus endometrium cancer group. On the other hand, endometrium cancer group elucidated a significant increase in seven metabolites namely; 5-dodecyldihydro-2 (3H)-furanone, oxymetholone, 5-Propyltridecane, 5-(2,6,6-Trimethyl-1-cyclohexen-1- yl)-4-penten-3-one, Propofol and Cis-9-tetradecenoic acid.

There was strong evidence that the first metabolite 5-dodecyldihydro-2 (3H)-furanone can change endocrine function, including several hormonal pathways, and can lead to cancers of the cervix, breast, lung, prostate, bladder, and brain. It has been demonstrated that 5-dodecyldihydro-2 (3H)-furanone contains estrogenic properties and can activate the estrogen receptor (ER).On the other hand, 5-dodecyldihydro-2 (3H)-furanone has anti-estrogenic properties by inhibiting aromatase, an enzyme that changes testosterone into E2, and by inducing cytochrome P450 1A1, which raises the metabolism of estrogen (E2). According to other research, 5-dodecyldihydro-2 (3H)-furanone inhibits the binding of dihydrotestosterone (DHT) to the androgen receptor (AR), hence having anti-androgenic effects. Follicle stimulating hormone, luteinizing hormone, prolaction, growth hormone, thyroid-stimulating hormone, and corticotrophin are all inhibited by 5-dodecyldihydro-2 (3H)-furanone. Changes in endocrine function may raise the risk of several malignancies (ovarian, uterine, and breast) and impact the development of hormone-responsive tissues. The actions of ∆9-THC on protein kinases provide evidence that 5-dodecyldihydro-2 (3H)-furanone modifies cell signaling pathways involved in cell cycle control.

There is prior evidence that oxymetholone is a well reported human carcinogen. In female rats, oxymetholone was thought to be linked to lung tumors, skin cancers, and endometrial cancer. The synthetic anabolic steroid oxymetholone shares structural similarities with the male hormone testosterone. Hypogonadism and delayed puberty are among the disorders of treatment with oxymetholone when given after ovulation, oxymetholone considerably reduces the length of the cycle's luteal phase, whilst ethylestrenol did the same for certain individuals. Analysis of oxymetholone's effects shows that it suppresses progesterone, follicle-stimulating hormone, and blood luteinizing hormone. Their antigonadotropic activity seems to be the cause of their ability to induce early menstruation and EC. If oxymetholone treatment is continued, women may develop male characteristics such as increased facial hair, male pattern baldness, breast swelling and changes in menstrual cycles [3].

Nonetheless, previous researchs revealed that propofol increased breast and gallbladder cancer cell invasion and proliferation. Propofol has been shown in numerous studies to trigger the spread of cancer cells in cervical cancer, breast cancer and colon cancer. Using atomic force microscopy, propofol altered the cervical cancer cells' cytoskeleton, perhaps revealing the molecular process by which propofol increased the ability to migrate. Propofol triggered the growth of tumors by increasing autophagic flux and causing autophagosomes to accumulate in cervical cancer cells. Propofol inhibited cancer cell apoptosis in addition to preventing breast cancer cells from adhering and migrating [8]. These results corroborate propofol's dual-edged properties as a tumor-suppressive and oncogenic agent in a variety of malignancies. Propofol activated Nrf2 at the transcriptional and translational levels, promoting cell invasion and proliferation in breast cancer in a dose-dependent and time-dependent way [24]. Accordingly, propofol caused cell migration via initiating the Nrf2 signaling pathway and, in part, by down regulating p53 expression in human breast cancer cells, which in turn caused cell proliferation. Furthermore, in order to elucidate the molecular mechanism behind the metastatic inhibitory effects of propofol in breast cancer, Li et al. [37] revealed that propofol hindered the capacity of cancer cells to invade and migrate and decreased MMP expression by decreasing NF-κB pathways. Propofol decreased apoptosis and increased cancer cell viability in cervical and endometrium cancer via blocking HSp70 ribosomal protein S6 kinase pathway, caspase-3, Bax and the mammalian target of rapamycin (mTOR) [9]. Propofol triggered Akt/mTOR, which prevented leukemia stem cells from maintaining self-renewing [10].

Lipid metabolism offers a network of pathways with feedback loops and crosstalk to join the elevated metabolic needs of cancer cells, researchers observed significant variation in fatty acids in various types of cancer for instance, hexadecanoic and octadecenoic may be elevated in endometrium cancer due to somatic mutation, DNA methylation abnormalities and regulation of transcription factors. Accordingly, octadecenoic acids reduce apoptosis and G0/G1 phase cell cycle arrest, greatly slowed cell proliferation, and triggered a number of tumor cells in a dose-dependent manner. Octadecenoic acids triggered the growth and DNA synthesis of human cervical cancer cells. Additionally, the cells appeared to be stimulated in entering the exponential phase of growth, indicating that octadecenoic acids have an effect on the early signals that lead to cell development [13].

5-Oxoprolinase levels were higher in ovarian cancer, more prevalent in tumor cells like bladder and nasopharyngeal cancer, and unaffected in colon cancer, indicating possible drawbacks of all-encompassing strategy. In the past, 5-oxoprolinase's main roles were linked to glutathione salvage, where the enzyme catalyzed the ATP-dependent cleavage of 5-oxoproline to produce glutamate. According to recent research on the γ-glutamyl cycle, 5-oxoproline levels might be a good indicator of nutritional quality, especially when it comes to the availability of glycine. Increased 5-oxoproline levels were seen when glycine became limiting, which promoted the intake of amino acids. This is in accordance with the finding that glycine deficiency causes a sharp rise in 5-oxoproline level and further induces EC [60].

In order to create a metabolic signature for endometrial cancer tumor surveillance, we used chromatographic analysis to identify free serum amino acids. Continuous monitoring revealed reduced glycine, serine, aspartic acid, and phenylalanine; leucine and isoleucine also showed altered levels. Nevertheless, a number of amino acids and their derivatives have been suggested as possible diagnostic biomarkers for EC in addition to lipid derivatives, which have been identified as potential biomarkers. Protein production depends on amino acids, which are also critical for cancer cells to progress. Furthermore, amino acids have been linked to the immunological and epigenetic regulatory processes in cancer cells and have the capacity to alter the redox balance [1]. Non-essential amino acids glycine and serine are important for the regulation of metabolism, including one-carbon metabolism, in addition to their role in protein synthesis. Type 2 diabetes and insulin resistance risk factors for endometrial cancer have been shown to have low plasma glycine level. Serine, which in this study was inversely correlated with the incidence of EC, is a component of the sphingolipid backbone. Ceramides, which are involved in cell migration, proliferation, and autophagy, can be produced from sphingomyelins, which account for involvement in carcinogenesis [17].

Indole-3-propionic acid (IPA) is an antioxidant recognized to guard against iron-induced oxidative abuse and carcinogenesis. Biological fluids including IPA (deamination product of tryptophan) effectively scavenges free radicals. GC–MS-based metabolomics revealed a number of metabolites, including heptanedioic acid, alanine, and phenylalanine, that are linked to treatment of endometrium cancer. Serum metabolites that include indole, indole-3-acetic acid, and indole-3-propionic acid are known as indole derivatives that are essential for preventing malignancies in the endometrium. Thus, RSV elevated tryptophan metabolites produced by the gut microbiota, could support its anticancer action against EC [1]. According to recent research, the gastrointestinal tract's bacterial fermentation produces short-chain fatty acids (SCFAs), which have been shown to have oncoprotective benefits against cervical cancer. Acetic acid, butyric acid, and propionic acid are the most widely well-known SCFAs; of these, propionic acid (PA) has been shown to cause apoptosis in cervical carcinoma (HeLa cells). Analysis using flow cytometry showed that PA causes reactive oxygen species (ROS), which causes the mitochondrial membrane to malfunction and suppresses the AKT/mTOR and NF-κB signaling pathways, which leads to autophagy. Additionally, PA boosted the sub-G1 cell population, which is indicative of cell death. Through a mechanism mediated by the stimulation of autophagy, PA reduces HeLa cell viability, suggesting a novel therapeutic approach for cervical cancer. SCFAs support hormone synthesis, and immunological responses, all of which contribute to maintaining human homeostasis [48].

Resvida (3,5,4'-trihydroxy-trans-stilbene) is derived from synthesis and recombinant technology although its existance in various plant-based sources. As RSV can bind to multiple enzymes and proteins of different pathways, which control gene expressions or modify the activity of other processes, it has been shown to manage a variety of cancer situations. Despite all of its positive effects on human health, it has certain drawbacks, such as low bioavailability, phase 2 metabolisms, liver and intestinal enzyme metabolism [47]. As an anti-cancer agent, RSV works incredibly by increasing the drug accumulation in specific location, nanocarriers have an advantage when it comes to delivering the medication to the intended site or, more generally, when it comes to increased bioavailability and therapeutic efficacy [55]. RSV structuraly like estradiol, a key component of estrogen, RSV is made up of phytoestrogen. RSV encompasses oxidative phosphorylation, insulin signaling, sterol biosynthesis, Kreb's cycle, glycolysis, and the electron transport chain. Because of its anti-inflammatory, anti-oxidant, anti-cancer, and neuroprotective qualities, RSV raises living standards [40]. To exhibit biological activity, RSV targets various proteins and enzymes, including ribonucleotide reductase, DNA polymerase, kinases, lipoxygenases, cyclooxygenases, P53, PTEN, mTOR and sirtuins [54].

Resveratrol may be a promising treatment for endometriosis because of its possible mechanisms, which include anti-proliferative, pro-apoptotic, anti-angiogenic, anti-oxidative stress, anti-invasive, and antiadhesive effects. By inhibiting cell proliferation, encouraging apoptosis, and decreasing invasiveness, RSV lessens the onset and progression of endometriosis. Given the documented upregulated expression of PCNA and downregulated expression of Ki-67 in implants, it is unclear whether resveratrol is effective in preventing the growth of ectopic endometrial lesions. Resveratrol treatment lowers the quantity of PCNA + and Ki-67 + endometrial cells in the rat model of peritoneal and mesenteric endometriosis, which slows the growth rate of endometriotic implants in comparison to the control group. Additionally, by preventing CD31 + endothelial cells from proliferating, resveratrol inhibits the development of new microvasculature in endometriotic implants [11].

By increasing caspase-3 cleavage in two cell lines, endometriotic epithelial cells (12Z) and endometrial stromal cells (St-T1b), RSV significantly lowers cell viability and triggers apoptosis. The ratio of matrix metalloproteinases (MMPs) to tissue inhibitors of matrix metalloproteinases (TIMPs) determines the invasiveness property. Additionally, by decreasing the gene expression ratio of MMP-2/TIMP-1 and inhibiting the mRNA expression of vascular endothelial growth factor (VEGF) and angiopoietin-1 (Ang-1), resveratrol reduces the capacity for invasion and angiogenesis. In addition to dramatically raising the expression levels of pro-apoptotic genes like Sirtuin-1 (SIRT1), Bax, caspase-3, and p53, and lowering the ratio of Bax/Bcl-2, RSV also lowers the level of nitric oxide (NO), a vasodilator and messenger molecule in the angiogenesis process.

Resveratrol intervention effectively reduces the gene expression ratio of Bax/Bcl-2, according to studies evaluating its impact on Bax and Bcl-2 gene expression within ectopic endometrial stromal cells (EcESCs), eutopic endometrial stromal cells (EuESCs), and NESCs. According to earlier research, resveratrol pretreatment reduces the level of the anti-apoptotic protein survivin, inhibits EcESCs' resistance to apoptosis, and speeds up apoptosis brought on by tumor necrosis factor-alpha (TNF-α) related apoptosis-inducing ligand (TRAIL). In contrast to the EuESCs and NESCs groups, RSV decreased the expression of IL-6 and IL-8 in EcESCs. In ectopic endometrium, overexpressed ERβ reduces TNF-α-induced apoptosis, increases IL-1β to stimulate inflammation, and improves EMT to promote invasion and adhesion.

At lower levels, RSV functions as an estrogen agonist; at higher levels, it functions as an antagonist. According to some research, resveratrol nanoparticles improve the bioavailability of resveratrol by increasing its uptake and sustained release in target cells, as well as its toxicity to these cells. Additionally, the absorption rate of resveratrol nanoparticles conjugated with polyethylene glycol is increased by seven times when compared to free resveratrol. Summerlin et al. suggested that methylated resveratrol produced by in vitro metabolic engineering (recombinant Escherichia coli) has higher biological activity than chemosynthesis. Methylation of resveratrol also increases its water solubility and bioavailability. Crucially, modifying the structure, bioavailability, and activity of resveratrol should be a priority during the drug research and development process [56].

Rats given resveratrol exhibited noticeably smaller endometriotic implant volumes. Superoxide dismutase and glutathione peroxidase activities in the rats' serum and tissue were found to significantly and dose-dependently increase following treatment. Similarly, compared to control animals, serum and tissue levels of catalase and malonyl dialdehyde were significantly higher. Following RSV treatment, there was also a significant decrease in histological scores and nuclear antigen expression levels in proliferating cells. In a variety of experimental models, RSV has been shown to suppress the generation of reactive oxygen species (ROS), inhibit the expression of cyclooxygenase-2 (COX-2), and inhibit the synthesis of prostaglandins [59].

Although the exact causes of cancer remain unknown, various theories include the cytoplasmic cell division, aberrant cell, and gene theories of RSV were reported. An enzyme in the cell nucleus called SIRT1is activated by RSV and is in charge of deacetylating histone and non-histone proteins [57]. Additionally, RSV is necessary to control several pathways that impact immunological function, inflammation, endothelial function, metabolism, cell survival and stress resistance. SIRT1 controls inflammation, cell cycle abnormalities, and aberrant metabolic control in cancer [63]. RSV prevents caspase-3 from simulating apoptosis mediated by interleukin 1. RSV prevented apoptosis Bcl-xL, Bcl-2, and TNF-α and decreased NF-κB-regulated gene products, including matrix metalloproteinase-2 and vascular endothelial growth factor. RSV suppresses the growth of EC cells and triggers apoptosis by blocking IGF-1R)/Wnt signaling pathways that activate p53. Numerous biological processes, including cell growth, survival, mobility, and differentiation and proliferation, are regulated by the PI3K/Akt/mTOR pathway. These pathways are an intriguing target for anti-cancer therapy because their components exhibit several aberrations during different tumor progression. When used in conjunction with other therapies, RSV suppresses the PI3K/Akt/mTOR pathways. In the ovarian cancer cell line (A2780 cell), RSV causes cell death, suppresses cell division, and modifies cell progression in EC. RSV causes autophagocytosis in EC cell A2780, and Bcl-2 and Bcl-xL can’t stop RSV's activity [51]. RSV possesses extremely ineffective oral doses because of its poor pharmacokinetic characteristics, which include solubility in an aqueous medium and high metabolization and excretion by the body [49]. RSV has a comparatively high oral absorption rate, but the liver and intestines quickly absorb the medication through first-pass metabolism. Demand has grown on pharmacokinetic enhancement of this dosage form. Nanotechnology is crucial when it comes to meeting the requirements of pharmacokinetic profiles, [2, 43]. Clinical studies have shown that liposomal anticancer drugs are less toxic and have better tolerance [43]. According to Jhaveri et al. [25], RSV-loaded liposomes exhibit stable, good loading efficiency and sustained release in vitro, which enhanced the anti-proliferation and apoptotic activities in U-87 MG cell line. 4-Carboxybutyl triphenylphosphonium bromide (TPP)-based liposomes demonstrated improved mitochondrial targeting and increased RSV activity in B16F10 cell lines [35]. Due to the targeting ligand attached to the surface of nanocarriers, favorable biodistribution, improved intracellular penetration, and long-lasting drug delivery in the blood, this improves in vivo efficiency. Traditional treatment techniques must give way to precision-driven strategies in light of the rapidly changing field of customized medicine. By combining cutting-edge molecular research with sophisticated liposomal drug delivery knowledge, our study explores the therapeutic effectiveness of liposomal reversterol in treating endometrium cancer [31]. According to our findings, Lip-RVS significantly affects nuclear morphology and cell cycle regulation, promoting early apoptosis and exhibiting prebiotic characteristics and autophagy mechanism via increasing drug bioavilability, retension time, skipping macrophage recognistion and targeting organs [48].

## Conclusion

Liposomal drug delivery system represented in liposomal reseveratrol could represent a prospective endometrium cancer adjuvant highlighting noninvasive metabolomics approach as a promising prognostic and diagnostic analysis combined with autophagy, cell survival and apoptotic pathways. The present srudy provides that liposomal reseveratrol significantly modulated autophagy biomarkers, such as mTOR/ERDj-4/p53/PTEN. Accordingly, multivariate data analyses showed that liposomal reseveratrol intervention may be a potential adjuvant for endometrial cancer therapy by restoring the level of metabolites back to normal.

### Study limitation

Difficality in handling OV as it may hazardous effects it required great caution. Animal study doesn’t highly mimic human study. Numbers of animals limited the studied parameters.

## Data Availability

No datasets were generated or analysed during the current study.

## Abbreviations

PTEN: Phosphatase and tensin homolog
PI3K: Phosphatidylinositol 3-kinase
AKT: Serine-threonine protein kinase-1
mTOR: Mechanistic target of rapamycin kinase
Cfos: AP-1 transcription factor subunit
HSP70: Heat shock protein-70
ERDJ: Endoplasmic reticulum Dnaj proteins
MDA: Malondialdehyde
EC: Endometrium cancer
OV: Oestradiol valerate

## References

[1] Ammar NM, Kadry MO, Abd Elkarim AS, Ibrahim RS, Sallam IE, El Gendy AEG, et al. Amaranthus spinosus Linn. extract as an innovative strategy to regulate biomarkers for ovarian hyperthecosis via circular RNA (hsa-circ-0001577): evidence from biochemical, metabolomics, histological, and phytochemical profiling. Food Sci Nutr. 2025;13(5):e70314. 10.1002/fsn3.70314. 40395717 10.1002/fsn3.70314 PMC12091212

[2] Annaji M, Poudel I, Boddu SHS, Arnold RD, Tiwari AK, Babu RJ. Resveratrol-loaded nanomedicines for cancer applications. Cancer Rep. 2021;4:e1353. 10.1002/cnr2.1353 PMC8222557 33655717

[3] Auerbach AD. Fanconi anemia and its diagnosis. Mutat Res. 2009;668(1–2):4–10. 10.1016/j.mrfmmm.2009.01.013. 19622403 10.1016/j.mrfmmm.2009.01.013 PMC2742943

[4] Bestvina CM, Fleming GF. Chemotherapy for endometrial cancer in adjuvant and advanced disease settings. Oncologist. 2016;21:1250–9. 10.1634/theoncologist.2016-0062. 27412393 10.1634/theoncologist.2016-0062 PMC5061541

[5] Bianco B, Barbosa CP, Trevisan CM, Laganà AS, Montagna E. Endometrial cancer: a genetic point of view. Transl Cancer Res. 2020;9(12):7706. 35117373 10.21037/tcr-20-2334 PMC8797944

[6] Bohaumilitzky L, von Knebel Doeberitz M, Kloor M, et al. Implications of hereditary origin on the immune phenotype of mismatch repair-deficient cancers: systematic literature review. J Clin Med. 2020;9:E1741. 10.3390/jcm9061741 PMC7357024 32512823

[7] Chen N, Karantza V. Autophagy as a therapeutic target in cancer. Cancer Biol Ther. 2011;11:157–68. 10.4161/cbt.11.2.14622. 21228626 10.4161/cbt.11.2.14622 PMC3230307

[8] Chen X, Li K, Zhao G. Propofol inhibits HeLa cells by impairing autophagic flux via AMP-activated protein kinase (AMPK) activation and endoplasmic reticulum stress regulated by calcium. Med Sci Monit. 2018;24:2339–49. 29667627 10.12659/MSM.909144 PMC5926273

[9] Chen F, Li M, Zhu X. Propofol suppresses proliferation and migration of papillary thyroid cancer cells by down-regulation of lncRNA ANRIL. Exp Mol Pathol. 2019;107:68–76. 30703346 10.1016/j.yexmp.2019.01.011

[10] Chen L, Wan Y, Liu Y, Li T. Propofol inhibits biological functions of leukaemia stem and differentiated cells through suppressing Wnt/beta-catenin and Akt/mTOR. Clin Exp Pharmacol Physiol. 2020;47(1):127–34. 31429973 10.1111/1440-1681.13167

[11] Cosco D, Paolino D, Maiuolo J, Marzio LD, Carafa M, Ventura CA, et al. Ultradeformable liposomes as multidrug carrier of resvida and 5-fluorouracil for their topical delivery. Int J Pharm. 2015;489:1–10. 25899287 10.1016/j.ijpharm.2015.04.056

[12] Dahiya V, Agam G, Lawatscheck J, Rutz DA, Lamb DC, Buchner J. Coordinated conformational processing of the tumor suppressor protein p53 by the Hsp70 and Hsp90 chaperone machineries. Mol Cell. 2019;74:816-830.e7. 31027879 10.1016/j.molcel.2019.03.026

[13] Datta S, Verma P, Dhara B, Kundu R, Maitra S, Mitra AK, et al. Interplay of precision therapeutics and MD study: *Calocybe indica’s* potentials against cervical cancer and its interaction with VEGF via octadecanoic acid. J Cell Mol Med. 2024;28(8):e18302. 38652115 10.1111/jcmm.18302 PMC11037404

[14] Daugaard M, Kirkegaard-Sorensen T, Ostenfeld MS, Aaboe M, Hoyer-Hansen M, Orntoft TF, et al. Lens epithelium-derived growth factor is an Hsp70-2 regulated guardian of lysosomal stability in human cancer. Cancer Res. 2007;67:2559–67. 17363574 10.1158/0008-5472.CAN-06-4121

[15] Denisenko TV, Pivnyuk AD, Zhivotovsky B. P53-autophagy-metastasis link. Cancers. 2018;10:148. 10.3390/cancers10050148. 29783720 10.3390/cancers10050148 PMC5977121

[16] Di Malta C, Cinque L, Settembre C. Transcriptional regulation of autophagy: mechanisms and diseases. Front Cell Dev Biol. 2019;7:114. 10.3389/fcell.2019.00114. 31312633 10.3389/fcell.2019.00114 PMC6614182

[17] Dossus L, Kouloura E, Biessy C, Viallon V, Siskos AP, Dimou N, et al. Prospective analysis of circulating metabolites and endometrial cancer risk. Gynecol Oncol. 2021;162(2):475–81. 34099314 10.1016/j.ygyno.2021.06.001 PMC8336647

[18] Du Toit A, Hofmeyr JS, Gniadek TJ, Loos B. Measuring autophagosome flux. Autophagy. 2018;14:1060–71. 10.1080/15548627.2018.1469590. 29909716 10.1080/15548627.2018.1469590 PMC6103398

[19] Elfiky AM, Ibrahim RS, Khattab AR, Kadry MO, Ammar NM, Shawky E. Exploring the therapeutic potential of marjoram (*Origanum majorana* L.) in polycystic ovary syndrome: insights from serum metabolomics, network pharmacology and experimental validation. BMC Complement Med Ther. 2025;25:67. 10.1186/s12906-025-04774-5. 39984989 10.1186/s12906-025-04774-5 PMC11846456

[20] Eritja N, Chen BJ, Rodríguez-Barrueco R, Santacana M, Gatius S, Vidal A, et al. Autophagy orchestrates adaptive responses to targeted therapy in endometrial cancer. Autophagy. 2017;4:608–24. 10.1080/15548627.2016.1271512 PMC5361596 28055301

[21] Gao Y, Han C, Huang H, Xin Y, Xu Y, Luo L, et al. Heat shock protein 70 together with its co-chaperone CHIP inhibits TNF-α induced apoptosis by promoting proteasomal degradation of apoptosis signal-regulating kinase1. Apoptosis. 2010;15:822–33. 20349136 10.1007/s10495-010-0495-7

[22] Graziele FdS, Samarina RW, Gisele M. Carboplatin: molecular mechanisms of action associated with chemoresistance. Braz J Pharm Sci. 2014;50:693–701. 10.1590/S1984-82502014000400004.

[23] Hassan S, Rizk M, El Sharkawi F, Badary O, Kadry M. The possible synergestic role of phytic acid and catechin in ameliorating the deteriorative biochemical effects induced by carbon tetrachloride in rats. J Appl Sci Res. 2007;3:1449–59.

[24] Jaura AI, Flood G, Gallagher HC, Buggy DJ. Differential effects of serum from patients administered distinct anaesthetic techniques on apoptosis in breast cancer cells in vitro: a pilot study. Br J Anaesth. 2014;113(suppl 1):i63–7. 25009197 10.1093/bja/aet581

[25] Jhaveri A, Deshpande P, Pattni B, Torchilin V. Transferrin-targeted, resveratrol-loaded liposomes for the treatment of glioblastoma. J Control Release. 2018;277:89–101. 29522834 10.1016/j.jconrel.2018.03.006 PMC5911193

[26] Kadry MO. Resveratrol-based nano-formulations as an emerging therapeutic strategy for ovarian carcinoma: autophagy stimulation and SIRT-1/Beclin/MMP-9/P53/AKT signaling. Cancer Nano. 2024;15:36. 10.1186/s12645-024-00274-2.

[27] Kadry MO, Abdel Megeed RM. Ubiquitous toxicity of mercuric chloride in target tissues and organs: impact of Ubidecarenone and liposomal-Ubidecarenone STAT 5A/ PTEN /PI3K/AKT signaling pathways. J Trace Elem Med Biol. 2022;74:127058. 10.1016/j.jtemb.2022.127058. 35952450 10.1016/j.jtemb.2022.127058

[28] Kadry MO, Abdel-Megeed RM. Bone marrow-derived mesenchymal stem cells mitigate caspase-3 and 8-hydroxy proline induced via β-adrenergic agonist in pulmonary injured rats. J Biochem Mol Toxicol. 2017. 10.1002/jbt.21913. 28266775 10.1002/jbt.21913

[29] Kadry MO, Abdel-Megeed RM. Titanium-nanostructured and PEGylated doxorubicin diminish chemotherapeutic resistance in 3-methylcholanthrene renal epithelial cell carcinoma via KRAS/FKBP5/P53/JAK2 signaling. Gene Expr. 2023;22(3):183–91. 10.14218/GE.2023.00069.

[30] Kadry MO, Abdel-Megeed RM. CRISPR-Cas9 genome and long non-coding RNAs as a novel diagnostic index for prostate cancer therapy via liposomal-coated compounds. PLoS ONE. 2024;19(5):e0302264. 10.1371/journal.pone.0302264. 38723038 10.1371/journal.pone.0302264 PMC11081254

[31] Kadry MO, Abdel-Megeed RM. Novel insights into SNORD-78 and miR-122-5P the predicted diagnostic indexes of lung cancer: drug-loaded liposome formulations competing methylcholanthrene-induced lung cancer. Cancer Nano. 2025;16:31. 10.1186/s12645-025-00331-4.

[32] Kadry MO, Ali HM. “Downregulation of HIF1-α, Smad-2, AKT, and Bax gene expression in sodium nitrite-induced lung injury via some antioxidants.” J Biochem Mol Toxicol. 2017. 10.1002/jbt.21909. 28266762 10.1002/jbt.21909

[33] Kadry MO, Ali HM. Impact of HIF1-α/TGF-β/Smad-2/Bax/Bcl2 pathways on cobalt chloride-induced cardiac and hepatorenal dysfunction. Future Sci OA. 2023;13(8):FSO874. 10.2144/fsoa-2023-0050. 10.2144/fsoa-2023-0050 PMC10445593 37621844

[34] Kai‐Chun C, Qu S, Chowdhury S, Noxon IC, Schonhoft JD, Plate L, et al. The endoplasmic reticulum HSP40 co-chaperone ERdj3/DNAJB11 assembles and functions as a tetramer. EMBO J. 2017;36:2296–309. 28655754 10.15252/embj.201695616 PMC5538767

[35] Kang JH, Ko YT. Enhanced subcellular trafficking of resvida using mitochondriotropic liposomes in cancer cells. Pharmaceutics. 2019;11:423. 31434345 10.3390/pharmaceutics11080423 PMC6722595

[36] Kumar A, Kurmi BD, Singh A, Singh D. Potential role of resvida and its nano-formulation as anti-cancer agent. Explor Target Antitumor Ther. 2022;3:643–58. 36338523 10.37349/etat.2022.00105 PMC9630550

[37] Li Q, Zhang L, Han Y, Jiang Z, Wang Q. Propofol reduces MMPs expression by inhibiting NF-kappaB activity in human MDA-MB-231 cells. Biomed Pharmacother. 2012;66(1):52–6. 22264881 10.1016/j.biopha.2011.10.006

[38] Lin Q, Chen H, Zhang M, Xiong H, Jiang Q. Knocking down FAM83B inhibits endometrial cancer cell proliferation and metastasis by silencing the PI3K/AKT/mTOR pathway. Biomed Pharmacother. 2019;115:108939. 10.1016/j.biopha.2019.108939. 31079003 10.1016/j.biopha.2019.108939

[39] Liu G, Ding W, Liu X, Mulder KM. c-Fos is required for TGFbeta1 production and the associated paracrine migratory effects of human colon carcinoma cells. Mol Carcinog. 2006;45(8):582–93. 16637060 10.1002/mc.20189

[40] Liu Q, Guan J, Sun Z, Shen X, Li L, Jin L, et al. Influence of stabilizer type and concentration on the lung deposition and retention of resvida nanosuspension-in-microparticles. Int J Pharm. 2019;569:118562. 31351178 10.1016/j.ijpharm.2019.118562

[41] Moore K, Brewer MA. Endometrial cancer: is this a new disease? Am Soc Clin Oncol. 2017;37:435–42. 10.14694/EDBK_175666. 10.1200/EDBK_175666 28561715

[42] Mrakovcic M, Fröhlich LF. p53-mediated molecular control of autophagy in tumor cells. Biomolecules. 2018;8(2):14. 10.3390/biom8020014. 29561758 10.3390/biom8020014 PMC6022997

[43] Nakhaei P, Margiana R, Bokov DO, Abdelbasset WK, Jadidi Kouhbanani MA, Varma RS, et al. Liposomes: structure, biomedical applications, and stability parameters with emphasis on cholesterol. Front Bioeng Biotechnol. 2021;9:705886. 34568298 10.3389/fbioe.2021.705886 PMC8459376

[44] Oliveira-Ferrer L, Rößler K, Haustein V, Schröder C, Wicklein D, Maltseva D, et al. c-FOS suppresses ovarian cancer progression by changing adhesion. Br J Cancer. 2014;110(3):753–63. 24322891 10.1038/bjc.2013.774 PMC3915133

[45] Olowofolahan AO, Tobih SE, Olorunsogo OO. Amelioration of oestradiol valerate-induced endometrial cancer in female rats by methanol fraction of *Mangifera indica* Linn. through modulation of oestrogen receptor signalling pathway. Indian J Physiol Pharmacol. 2021;65(2):94–102.

[46] Park S-H, Baek K-H, Shin I, Shin I. Subcellular Hsp70 inhibitors promote cancer cell death via different mechanisms. Cell Chem Biol. 2018;25:1242-1254.e8. 10.1016/j.chembiol.2018.06.010. 30057298 10.1016/j.chembiol.2018.06.010

[47] Pezzuto JM. Resveratrol: twenty years of growth, development and controversy. Biomol Ther (Seoul). 2019;27:1–14. 30332889 10.4062/biomolther.2018.176 PMC6319551

[48] Pham CH, Lee JE, Yu J, Lee SH, Yu KR, Hong J, et al. Anticancer effects of propionic acid inducing cell death in cervical cancer cells. Molecules. 2021;26(16):4951. 34443546 10.3390/molecules26164951 PMC8399869

[49] Ren B, Kwah MX, Liu C, Ma Z, Shanmugam MK, Ding L, et al. Resvida for cancer therapy: challenges and future perspectives. Cancer Lett. 2021;515:63–72. 34052324 10.1016/j.canlet.2021.05.001

[50] Rizk MZ, Ali SA, Kadry MO, Fouad GI, Kamel NN, Younis EA, et al. C-reactive protein signaling and chromosomal abnormalities in nanotoxicity induced via different doses of TiO_2_ (80 nm) boost liver function. Biol Trace Elem Res. 2020;198(1):157–67. 32016825 10.1007/s12011-020-02030-0

[51] Rizvi SAA, Saleh AM. Applications of nanoparticle systems in drug delivery technology. Saudi Pharm J. 2018;26:64–70. 29379334 10.1016/j.jsps.2017.10.012 PMC5783816

[52] Rosenzweig R, Nillegoda NB, Mayer MP, Bukau B. The Hsp70 chaperone network. Nat Rev Mol Cell Biol. 2019;20:665–80. 10.1038/s41580-019-0133-3. 31253954 10.1038/s41580-019-0133-3

[53] Shamseldean MSM, Allam SFM, Rezk MZA. Chemical components and field applications of essential oils to prevent date palm infections by Rhynchophorus ferrugineus (Coleoptera: Curculionidae) an invasive species in Bahariya Oasis, Egypt. Biosci Res. 2022;19(4):2223–41.

[54] Sharifi-Rad J, Quispe C, Alfred MA, Anil N, Lombardi N, Cinquanta L, et al. Current trends on resvidabioactivities to treat periodontitis. Food Biosci. 2021;42:101205.

[55] Springer M, Moco S. Resvidaand its human metabolites-effects on metabolic health and obesity. Nutrients. 2019;11:143. 30641865 10.3390/nu11010143 PMC6357128

[56] Tao J, Chen Y, Gu X, Miao M, Hu D, Zhou H, et al. Review of the potential therapeutic effects and molecular mechanisms of resveratrol on endometriosis. Int J Women’s Health. 2023. 10.2147/IJWH.S404660. 10.2147/IJWH.S404660 PMC10187648 37200624

[57] Thiel G, Rössler OG. Resvida regulates gene transcription via activation of stimulus-responsive transcription factors. Pharmacol Res. 2017;117:166–76. 28012964 10.1016/j.phrs.2016.12.029

[58] Urick ME, Bell DW. Clinical actionability of molecular targets in endometrial cancer. Nat Rev Cancer. 2019;19:510–21. 10.1038/s41568-019-0177-x. 31388127 10.1038/s41568-019-0177-x PMC7446243

[59] Yavuz S, Aydin NE, Celik O, Yilmaz E, Ozerol E, Tanbek K. Resveratrol successfully treats experimental endometriosis through modulation of oxidative stress and lipid peroxidation. J Cancer Res Ther. 2014;10(2):324–9. 10.4103/0973-1482.136619. 25022386 10.4103/0973-1482.136619

[60] Yilin L, Hyde AS, Simpson MA, Barycki JJ. Chapter two - emerging regulatory paradigms in glutathione metabolism. In: Townsend DM, Tew KD, editors. Advances in cancer research, vol. 122. Academic Press; 2014. p. 69–101. 10.1016/B978-0-12-420117-0.00002-5 PMC4515967 24974179

[61] Zarema A, Armeev GA, Kanevskiy LM, Kovalenko EI, Sapozhnikov AM. HSP70 multi-functionality in cancer. Cells. 2020;9(3):587. 10.3390/cells9030587. 32121660 10.3390/cells9030587 PMC7140411

[62] Zhang H, Amick J, Chakravarti R, Santarriaga S, Schlanger S, McGlone C, et al. A bipartite interaction between Hsp70 and CHIP regulates ubiquitination of chaperoned client proteins. Structure. 2015;23:472–82. 10.1016/j.str.2015.01.003. 25684577 10.1016/j.str.2015.01.003 PMC4351142

[63] Zhao M, Ko SY, Garrett IR, Mundy GR, Gutierrez GE, Edwards JR. The polyphenol resvida promotes skeletal growth in mice through a sirtuin 1-bone morphogenic protein 2 longevity axis. Br J Pharmacol. 2018;175:4183–92. 30125963 10.1111/bph.14477 PMC6177622

[64] Zhu H, Luo H, Zhang W, Shen Z, Hu X, Zhu X. Molecular mechanisms of cisplatin resistance in cervical cancer. Drug Des Devel Ther. 2016;7:1885–95. 10.2147/DDDT.S106412. 10.2147/DDDT.S106412 PMC4907638 27354763

